# *In silico* analysis of single nucleotide polymorphisms (SNPs) in human FOXC2 gene

**DOI:** 10.12688/f1000research.10937.2

**Published:** 2017-10-09

**Authors:** Mohammed Nimir, Mohanad Abdelrahim, Mohamed Abdelrahim, Mahil Abdalla, Wala eldin Ahmed, Muhanned Abdullah, Muzamil Mahdi Abdel Hamid

**Affiliations:** 1 Soba Center for Audit and Research, Soba University Hospital, University of Khartoum, Khartoum, 11111, Sudan; 2 Department of Human Anatomy, Ahfad University for Women, Khartoum, 11111, Sudan; 3 Department of Internal Medicine, Faculty of Medicine, University of Khartoum, Khartoum, 11111, Sudan; 4 Institute of Endemic Diseases, University of Khartoum, Khartoum, 11111, Sudan

**Keywords:** Primary lymphedema, FOXC2, SNP, In silico, Bioinformatics, miRNA

## Abstract

**Introduction**: Lymphedema is an abnormal accumulation of interstitial fluid, due to inefficient uptake and reduced flow, leading to swelling and disability, mostly in the extremities. Hereditary lymphedema usually occurs as an autosomal dominant trait with allelic heterogeneity.

**Methods**: We identified single nucleotide polymorphisms (SNPs) in the FOXC2 gene using dbSNP, analyzed their effect on the resulting protein using VEP and Biomart, modelled the resulting protein using Project HOPE, identified gene – gene interactions using GeneMANIA and predicted miRNAs affected and the resulting effects of SNPs in the 5’ and 3’ regions using PolymiRTS.

**Results**: We identified 473 SNPs - 429 were nsSNPs and 44 SNPs were in the 5’ and 3’ UTRs. In total, 2 SNPs - rs121909106 and rs121909107 - have deleterious effects on the resulting protein, and a 3D model confirmed those effects. The gene – gene interaction network showed the involvement of FOXC2 protein in the development of the lymphatic system. hsa-miR-6886-5p, hsa-miRS-6886-5p, hsa-miR-6720-3p, which were affected by the SNPs rs201118690, rs6413505, rs201914560, respectively, were the most important miRNAs affected, due to their high conservation score.

**Conclusions**: rs121909106 and rs121909107 were predicted to have the most harmful effects, while hsa-miR-6886-5p, hsa-miR-6886-5p and hsa-miR-6720-3p were predicted to be the most important miRNAs affected. Computational biology tools have advantages and disadvantages, and the results they provide are predictions that require confirmation using methods such as functional studies.

## Introduction

Lymphedema is an abnormal accumulation of interstitial fluid, due to inefficient uptake and reduced flow, leading to swelling and disability, which mostly affects the extremities. It can be divided into primary and secondary lymphedema according to the underlying cause. Primary or hereditary lymphedema results from genetic damage, while secondary or acquired lymphedema is caused by lymphatic system malfunction, resulting from trauma, including surgery, radiotherapy, tumors, or infections (for example, parasitic infections) ^1^.

Hereditary lymphedema usually occurs as an autosomal dominant trait with allelic heterogeneity. The most common type of primary hereditary lymphedema, Milroy disease, can develop due to mutations in the vascular endothelial growth–factor receptor-3 gene (VEGFR-3; FLT4) ^2^.

Forkhead box (Fox) proteins are a family of transcription factors (TFs) that play a key role in cell development, cell cycle regulation, and other important biological processes ^3^. FOXC2, was first identified as a transcription factor (TF) that plays a key role in the morphogenesis of the cardiovascular system ^4^. Further studies revealed that FOXC2 was involved in lymphatic vascular development. In both humans and mice, FOXC2 is expressed in large amounts in the developing lymphatic vessels and in adult lymphatic valves ^5^. FOXC2-deficient mice were demonstrated to have abnormal lymphatic patterning and failure to form lymphatic valves, which reveals the critical role of FOXC2 in lymphatic vascular development ^6^. Truncating and missense mutations of FOXC2 have been discovered in patients with late-onset lymphedema (hereditary lymphedema II; OMIM 153200), often associated with distichiasis (double row of eyelashes), and sometimes ptosis (Lymphedema Distichiasis Syndrome [LDS]; OMIM 153400), and/or yellow nails (OMIM 153300) ^7– 9^. LDS patients develop defects characterized by lymphatic and venous blood reflux, which means failure and/or absence of lymphatic and venous valves ^10, 11^. On a molecular level, FOXC2 DNA binding sites are enriched in nuclear factor of activated T-cells 1 (NFATC1) consensus sequences, and the two TFs work in tandem during lymphatic vascular morphogenesis ^12^. FOXC2 controls the expression of proteins that are essential for lymphatic valve development, such as connexins. Thus, adequate control of their activity is extremely important for proper lymphatic vessel development and function ^13^.

The aetiology governing the phenotypic variability remains unclear ^14^. Lymphatic malformations could also result from slightly mutated germline alleles, which are difficult to access (and so to identify), mutations in regulatory regions of the DNA, epigenetic changes, or a combination of the three. Epigenome sequencing and whole exome sequencing may be needed for in-depth study of these mutations. The part genetics play in the development of lymphatic anomalies is highly complex, shown by the discovery of 23 mutated human genes ^13^.

Few studies addressing FOXC2 mutations from a bioinformatics point of view have been published. Out of those, none have specifically focused on single nucleotide polymorphisms (SNPs). SNPs may affect codons of amino acids located at the forkhead active domain of the protein or other sites and may severely affect the function of the TFs. Furthermore, SNPs are feasible and cost effective to study using *in silico* analysis via available bioinformatics tools.

This study aimed to analyze all SNPs in the human FOXC2 gene and predict their effect on the structure, function, stability and regulation of its respective protein. The results of our study can be used in population studies to screen patients with hereditary lymphedema, and more importantly in phenotypic variations in lymphatic malformations of affected individuals.

## Methods

### Mining the database for SNPs

We selected the National Center for Biotechnology Information (NCBI) database, dbSNP ( http://www.ncbi.nlm.nih.gov/projects/SNP) for the retrieval of SNPs and their related protein sequence of FOXC2 gene. We used “FOXC2” as our search term and used filters to narrow down our search results into two categories: 3’ + 5’ UTR SNPs only and all SNPs in FOXC2, except for those in the 3’ + 5’ UTR. This gene was chosen because it’s the one that is known to be associated with LDS, used for our computational analysis. It should be noted that dbSNP has its caveats, like high false positive rates, but this was partially overcome by using several SNP effect prediction tools (as outlined below).

### Evaluation of coding SNPs

We chose three complementary algorithms for functional impact prediction of nsSNPs: Sorting Intolerant From Tolerant (SIFT; http://sift.bii.a-star.edu.sg/), Polymorphism Phenotyping (PolyPhen; http://genetics.bwh.harvard.edu/pph/) and CONsensus DELeteriousness (Condel; http://bg.upf.edu/fannsdb/) ^15– 17^.

SIFT version 2.0 was used to distinguish between tolerant and intolerant coding mutations, and is used to predict whether an amino acid substitution in a protein will have a phenotypic effect. SIFT is based on the premise that protein evolution is correlated with protein function. Variants that occur at conserved alignment positions are expected to be tolerated less than those that occur at diverse positions. PolyPhen is a computational tool for identification of potentially functional nsSNPs. Predictions are based on a combination of phylogenetic, structural and sequence annotation information characterizing a substitution and its position in the protein. These algorithms can possibly identify pathogenic SNPs, and using three algorithms increases the robustness of the results by avoiding false negatives and positives.

We uploaded the SNP accession numbers of all the SNPs into VEP ( http://www.ensembl.org/Tools/VEP) ^18^ and enabled “SIFT”, “PolyPhen” and “Condel” to retrieve the corresponding predictions of the functional significance of each SNP from all of the three algorithms. We repeated this step using Biomart ( http://www.ensembl.org/biomart/martview/) ^19^, which is similar to VEP and part of Ensembl. It provides a SIFT and PolyPhen prediction and score, like VEP, but it does not provide a Condel prediction. We took the SNPs predicted by both databases to be deleterious and pathogenic (2 SNPs; rs121909106 and rs121909107) to perform the next steps in the analysis.

### Predicting the molecular phenotypic effects of deleterious SNPs

Project HOPE ( http://www.cmbi.ru.nl/hope/) is an online web-server used to search protein 3D structures by collecting structural information from a series of sources. We entered the two SNPs (rs121909106 and rs121909107) that we obtained from the last step together with the primary structure of the FOXC2 protein (obtained from the Protein Data Bank http://www.rcsb.org/pdb/explore/explore.do?structureId=1D5V) into HOPE ^20^. HOPE lets you choose the amino acid that was affected by the mutation and allows you to change that specific amino acid and then analyse the resulting change in the 3D (tertiary/quaternary) structure and outputs the change predicted, plus the explanation of such a change (in both structure and function of the protein).

### Outlining gene – gene interactions

Gene-gene interactions were studied to highlight candidate genes that could possibly be associated with LDS, especially if haplotypes were to be studied in the future. GeneMANIA ver. 3.1.2.8 ( http://genemania.org/) finds other genes that are related to a set of input genes, using a very large set of functional association data ^21^. It was chosen for its speed and accuracy in prediction of gene-gene interactions, in addition to its relatively frequent version updates. Association data include protein and genetic interactions, pathways, co-expression, co-localization and protein domain similarity. We entered FOXC2 as our query gene and the website generated a network of genes along with their gene-gene interactions, according to gene ontology terms.

### Characterization of SNPs in 3’ and 5’ untranslated regions

PolymiRTS v3.0 ( http://compbio.uthsc.edu/miRSNP/) is an integrated platform for analyzing the functional impact of genetic polymorphisms in miRNA seed regions and miRNA target sites ^22^. We entered a second set of SNPs (containing only 5’ and 3’ SNPs) and acquired a list of the miRNAs affected by these mutations. The affected miRNAs might lead to a decrease/increase of the expression of FOXC2.

**Figure 1.**
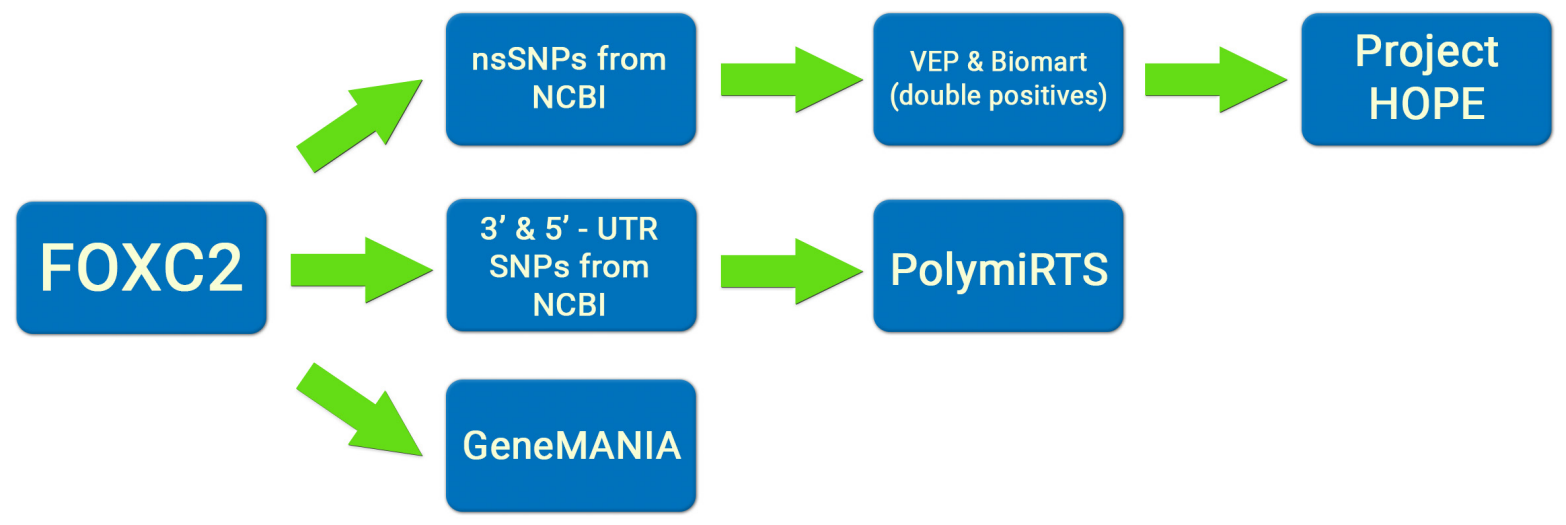
Flow chart of the analysis process.

The analysis steps performed are summarized in Figure 1.

## Results

### Predictions of deleterious and damaging coding nsSNPs

SNP information for FOXC2 was retrieved from dbSNP. For our investigations, we selected SNPs in coding and UTR (5’and 3’) regions. Among the 473 SNPs, 429 were nsSNPs and 44 SNPs were in the 5’ and 3’ UTRs of FOXC2.

### VEP and Biomart

We found two SNPs, rs121909106 and rs121909107, which correspond to S125L and R121H, to have significant deleterious effect on the structure and function of FOXC2 protein. We summarized the information of the two SNPs predicted by the two databases in Table 1.

**Table 1.** Results of analysis by VEP and BioMart.

Accession number		rs121909106	rs121909107
**Analysis** **by VEP**	**Location**	16:86567709	16:86567697
	**Allele**	T	A
	**Position**	125	121
	**Amino Acids**	S/L	R/H
	**Codons**	tCg/tTg	cGc/cAc
	**SIFT**	Deleterious (0)	Deleterious (0)
	**PolyPhen**	Probably damaging (0.994)	Probably damaging (0.998)
	**Condel**	Deleterious (0.997)	Deleterious (0.999)
	**Clinical Significance**	Pathogenic	Pathogenic
**Analysis** **by BioMart**	**Chromosome**	16	16
	**Position (bp)**	86567709	86567697
	**Alleles**	C/T	G/A
	**Clinical significance**	Pathogenic	Pathogenic
	**PolyPhen prediction**	Probably damaging (0.994)	Probably damaging (0.998)
	**SIFT prediction**	Deleterious (0)	Deleterious (0)
	**Condel**	Deleterious (0.997)	Deleterious (0.999)

### Project HOPE

**Figure 2.**
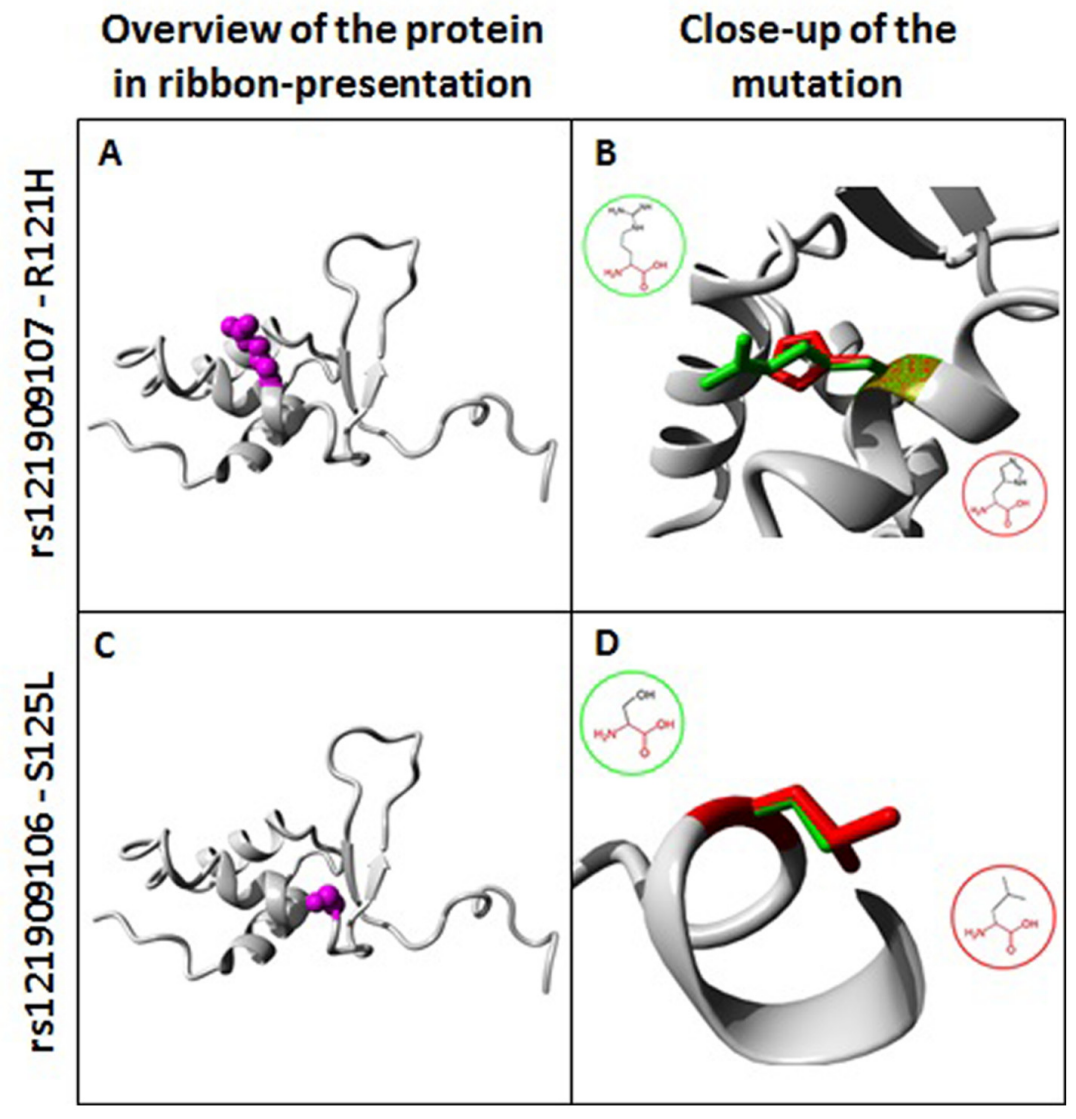
3D representation of rs121909107 and rs121909106 and their effect of FOXC2. Overview shows the protein in grey and the affected amino acid in purple, the close-up shows the side chains of both the wild-type (Arginine) and the mutant residue (Histidine) at position 121 and colored green and red respectively. For rs121909106; overview shows the protein in grey and the affected amino acid in purple, the close-up shows the side chains of both the wild-type (Serine) and the mutant residue (Leucine) at position 125 are shown and colored green and red, respectively.

Figure 2 shows a 3D model of rs121909107 and rs121909106 and the spatial effects of each on the respective domains they are a part of, in addition to their effects on neighbouring domains.

### GeneMANIA

**Figure 3.**
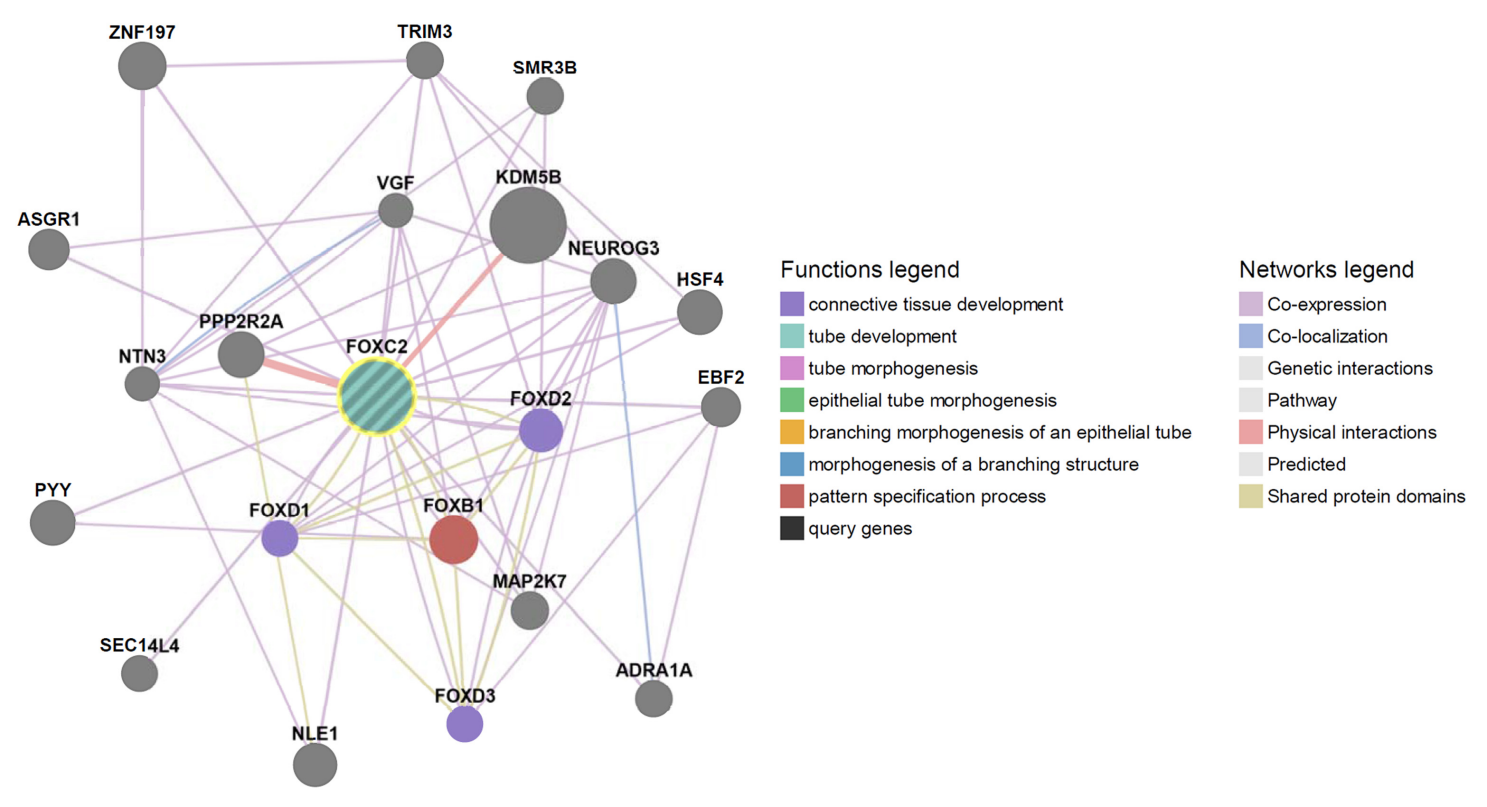
Gene-gene interaction network of FOXC2 with paths colored according to their functions.

Figure 3 shows the gene-gene interactions of FOXC2. The most important interactions are with FOXB1, FOXD1, FOXD2 and FOXD3 ( Figure 3) (additional data in Supplementary material).

### PolymiRTS

Table 2– Table 4 show the effect that SNPs in the 3’ and 5’ region of FOXC2 might have, along with the conservation score (CS) and context+ score of the site in question. The higher the CS, the more profound the effect of the SNP is predicted to be. In addition, the higher the context+ score, the higher the likelihood of a change (disruption or creation) occurring in the miRNA target site.

**Table 2.** SNPs and INDELs in miRNA target sites.

Location	dbSNP ID	Wobble base pair	Ancestral allele	Allele	miR ID	Conservation	miRSite	Function Class	Exp Support	context+ score change
86602454	rs200353178	N	C	C	hsa-miR-1247-3p hsa-miR-4449 hsa-miR-4532	2 3 3	cgtgTCCCGGGAc cgtgtcCCGGGAC cgtgtCCCGGGAc	D D D	N N N	No Change No Change No Change
				T	hsa-miR-4740-3p	2	cgtgTCTCGGGAc	C	N	No Change
86602489	rs3751795	N	C	C	hsa-miR-4747-5p hsa-miR-5196-5p	2 2	gcttcgCTTCCCA gcttcgCTTCCCA	D D	N N	-0.054 -0.057
				T	hsa-miR-3153 hsa-miR-4668-5p hsa-miR-6733-5p hsa-miR-6739-5p	2 2 2 2	gcttcgTTTCCCA gcttcgTTTCCCA gcttcgTTTCCCA gcttcgTTTCCCA	C C C C	N N N N	-0.024 -0.022 -0.021 -0.027
86602513	rs142773766	N	C	C	hsa-miR-4288 hsa-miR-632	2 2	acCAGACAAttaa acCAGACAAttaa	D D	N N	-0.117 -0.117
				T	hsa-miR-1298-3p hsa-miR-3126-3p	3 3	aCCAGATAattaa aCCAGATAattaa	C C	N N	-0.114 -0.132
Location		SNP location in the mRNA transcript. It is a zero-based number.								
SNPID		Link to dbSNP.								
Wobble Pair		Whether the SNP can form a G:U wobble basepair with the miRNA. Y: Yes; N: No.								
Ancestral Allele		If applicable, the ancestral allele is denoted.								
Allele		Two alleles of the SNP in the mRNA transcript.								
miRID		Link to miRBase.								
Conservation		Occurrence of the miRNA site in other vertebrate genomes in addition to the query genome. By clicking the hyperlink, the users can examine the genomes in which this miRNA target site occurs.								
FuncClass		**D**: The derived allele disrupts a conserved miRNA site (ancestral allele with support >= 2). **N**: The derived allele disrupts a nonconserved miRNA site (ancestral allele with support < 2). **C**: The derived allele creates a new miRNA site. **O**: The ancestral allele can not be determined.								
miRSite		Sequence context of the miRNA site. Bases complementary to the seed region are in capital letters and SNPs are highlighted in red.								
ExpSupport		**LT**: The miRNA-mRNA interaction is supported by a low-throughput experiment (e.g., luciferase reporter assay or Western blot). **HT**: The miRNA-mRNA interaction is supported by a high-throughput experiment (e.g., microarray or pSILAC). **LTL**: The miRNA targeting the specific location is supported by a low-throughput experiment (e.g., allelic imbalance sequencing). **HTL**: The miRNA targeting the specific location is supported by a high-throughput experiment (e.g., HITS-CLIP).								
Context+ score change		Context+ scores predict the binding of a miRNA to the entire 3'-UTR by summing over contributions made by individual sites within the 3'-UTR that have perfect sequence complementarity to the miRNA seed region. The change the differences in context+ scores between the reference and derived alleles for each SNP or INDEL in putative miRNA target sites. A more negative value of the context+ score difference indicates an increased likelihood that the miRNA targeting is disrupted or newly created by the mutation in the target sites								

**Table 3.** Target sites disrupted by single nucleotide polymorphisms (SNPs) and INDELs in miRNA seeds.

Location	miR ID	dbSNP ID	miR seed	Allele	Wobble base pair	miRSite	Conservation	context+score change
**86602466**	hsa-miR-6889-5p	rs146254801	C[G/A]GGGAG	G/A	1	CUCCCCG	2	-0.182
**86602496**	hsa-miR-5090	rs3823658	C[G/A]GGGCA	G/A	1	GCCCCGA	2	-0.227
**86602496**	hsa-miR-6727-5p	rs202175375	U[C/T]GGGGC	C/T	0	GCCCCGA	2	-0.291
**86602466**	hsa-miR-6777-5p	rs56155608	[C/T]GGGGAG	C/T	0	CUCCCCG	2	-0.144

**Table 4.** Target sites created by single nucleotide polymorphisms (SNPs) and INDELs in miRNA seeds.

Location	miR ID	dbSNP ID	miR Seed	Allele	Wobble base pair	miRSite	Conservation	context+score change
**86602524**	hsa-miR-6886-5p	rs201118690	[C/T]CGCAGG	C/T	0	CUGCAGA	7	-0.074
**86602524**	hsa-miR-6886-5p	rs6413505	C[C/T]GCAGG	C/T	0	CUGCAGA	7	-0.074
**86602530**	hsa-miR-6720-3p	rs201914560	GCG[C/T]CUG	C/T	0	AGACGCA	6	-0.108
**86602497**	hsa-miR-4472	rs28655823	GU[G/C]GGGG	G/C	0	CCCCGAC	2	-0.244
**86602497**	hsa-miR-4472	rs202127912	GU[TG/-]GGGG	TG/-	0	CCCCGAC	2	-0.244

SNPs used for PolymiRTS analysis Click here for additional data file. Copyright: © 2017 Nimir M et al. 2017 Data associated with the article are available under the terms of the Creative Commons Zero "No rights reserved" data waiver (CC0 1.0 Public domain dedication).

SNPs used for other analyses Click here for additional data file. Copyright: © 2017 Nimir M et al. 2017 Data associated with the article are available under the terms of the Creative Commons Zero "No rights reserved" data waiver (CC0 1.0 Public domain dedication).

## Discussion

This study aimed to analyze the SNPs identified in the FOXC2 gene. We found a total of 473 SNPs, 2 of which were predicted to adversely affect the function of the resulting protein. One mutation leads to translation of a histidine instead of an arginine at position 121, and the mutant residue is located near a highly conserved region ^20^. The mutation was reported previously in a patient suffering from lymphedema-distichiasis syndrome by Berry *et al.* (2005), and the effects that the R121H mutation would have on the DNA-binding part of the forkhead domain (FHD) of FOXC2 was predicted. It was predicted that R121H would impair FOXC2 protein’s ability to bind DNA and act as a transcription activator, and this was confirmed by biochemical studies. Also, the mutation resulted in the mislocalization of FOXC2. Berry *et al.* (2005) determined that the mutation results in a non-functional protein and that this leads to hereditary LDS ^23^. The residue affected by rs121909107 is part of an inter pro-domain named Fork Head Domain Conserved Site 2 (Interpro, IPR030456), and it is annotated with the following Gene-Ontology (GO) terms to indicate its function: sequence-specific DNA binding (GO:0043565) and sequence-specific DNA binding transcription factor activity (GO:0003700).

The change of a serine into a leucine at position 125 means that a nonpolar amino acid will be replaced with a polar one. The original wild-type residue and newly introduced mutant residue differ in their electrochemical properties. The mutation results in incorporating an amino acid with a different level of hydrophobicity, this will affect hydrogen bond formation, and the mutant residue is located near a highly-conserved region ^20^. This mutation matches a previously described variant in affected members of families with lymphedema-distichiasis syndrome, previously reported by Mangion *et al.* ^24^ and Bell *et al.* ^7^. The mutation was not identified in 100 normal chromosomes. This mutation results in a wide range of phenotypes from minimal distichiasis to severe lymphoedema and congenital heart disease ^7^. The residue affected by rs121909106 is part of an inter prodmain named Winged Helix-Turn-Helix DNA-Binding Domain (IPR011991) and it is annotated with the following GO terms to indicate its function: nucleic acid binding (GO:0003676) and nucleic acid binding transcription factor activity (GO:0001071). No coordination was found between the two SNPs, and they are not known to belong to any haplotype.

Our results from GeneMANIA showed that FOXC2 interacts with a lot of genes ( Figure 3), mainly functioning to control connective tissue, tube and epithelial tissue development. These results were proved important by Mangion *et al.* ^24^ and Bell *et al.* ^7^, which showed that mutations in FOXC2 lead to developing LDS.

PolymiRTS results showed SNPs and INDELs in miRNA target sites: target sites disrupted by SNPs and INDELs in miRNA seeds and target sites created by SNPs and INDELs in miRNA seeds ( Table 2– Table 4). Three miRNAs that are worth noting are hsa-miR-6886-5p (with a CS of 7), hsa-miR-6886-5p (with a CS of 7) and hsa-miR-6720-3p (with a CS of 6), which are affected by the SNPs rs201118690, rs6413505, rs201914560, respectively. This points to the possibility that the areas affected by those SNPs have an evolutionary important function.

## Conclusions

In conclusion, there are many SNPs that affect FOXC2 gene; some are predicted to be harmful, such as rs121909106 and rs121909107, but most are not. miRNAs were affected by SNPs in the 3’ and 5’ untranslated regions of FOXC2 gene and three are noteworthy, hsa-miR-6886-5p, hsa-miR-6886-5p and hsa-miR-6720-3p, due their high conservation score.

Computational biology tools are very powerful, especially when provided with good data and used by experts. However, bioinformatics tools have their limitations; most importantly the fact that their results are but predictions, meaning that the information they provide us with requires confirmation using various methods such as functional studies.

## Data availability

The data referenced by this article are under copyright with the following copyright statement: Copyright: © 2017 Nimir M et al.

Data associated with the article are available under the terms of the Creative Commons Zero "No rights reserved" data waiver (CC0 1.0 Public domain dedication). 

Dataset 1: SNPs used for PolymiRTS analysis. doi, 10.5256/f1000research.10937.d153064 ^25^

Dataset 2: SNPs used for other analyses. doi, 10.5256/f1000research.10937.d153065 ^26^

## Supplementary material

Supplementary File 1: GeneMANIA network as an Excel file.

Supplementary File 2: Print-out of the full report obtained from the GeneMANIA website for FOXC2 gene.
