# Supplementary material for: In silico analysis of single nucleotide polymorphisms (SNPs) in human FOXC2 gene
Source: F1000Res. 2017 Oct 9;6:243. Originally published 2017 Mar 9. [Version 2] doi: 10.12688/f1000research.10937.2 (PMC5814747; doi:10.12688/f1000research.10937.2)
Supplement: Supplementary file 4 [file f1000research-6-13959-s0003.tgz › 936de9e9-0ab0-4dbb-9e68-74f07b3213a7.pdf]

GeneMANIA

Created on: 2 January 2016 00:24:09
Last database update: 1 June 2014 20:00:00
Application version: 3.1.2.8

Report of GeneMANIA search

Network image

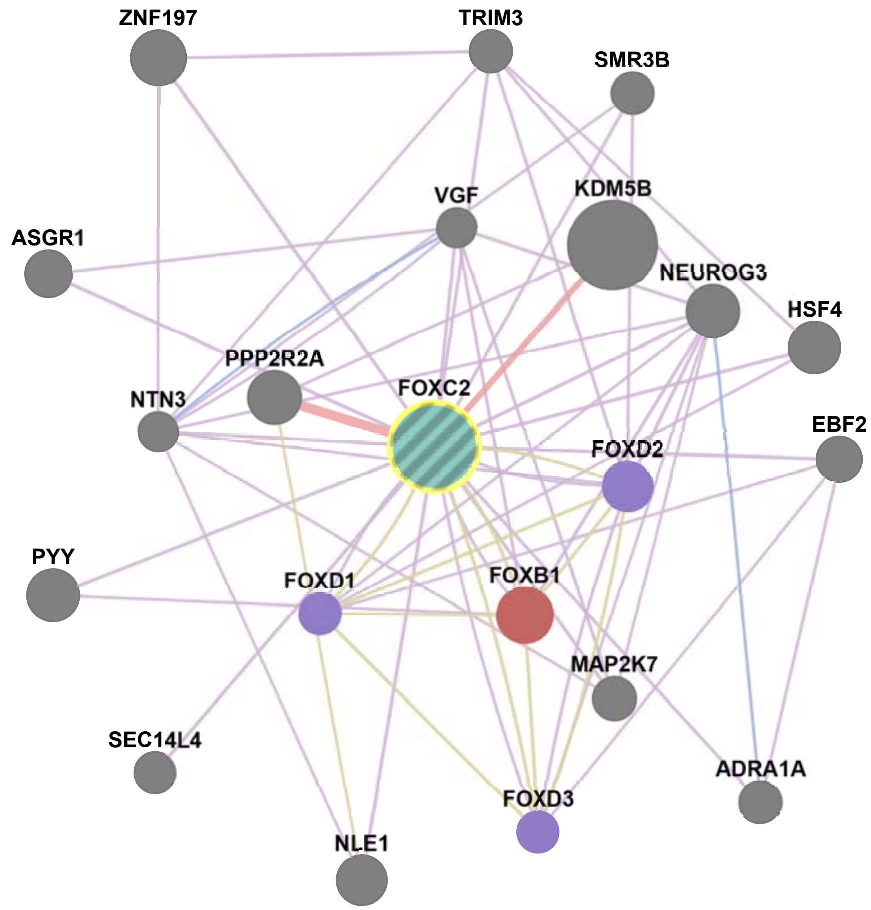

Functions legend

- connective tissue development
- tube development
- tube morphogenesis
- epithelial tube morphogenesis
- branching morphogenesis of an epithelial tube
- morphogenesis of a branching structure
- pattern specification process
- query genes

Networks legend

- Co-expression
- Co-localization
- Genetic interactions
- Pathway
- Physical interactions
- Predicted
- Shared protein domains

Search parameters

Organism: H. sapiens (human)

Genes: FOXC2

Networks:

Attributes:

Co-expression:
Alizadeh-Staudt-2000 Arijs-Rutgeerts-2009 Bahr-Bowler-2013 Bild-Nevins-2006 B Boldrick-Relman-2002 Burington-Shaughnessy-2008 Chen-Brown-2002 Cheok-Evans-2003 Gysin-McMahon-2012 Innocenti-Brown-2011 Kang-Willman-2010 Mallon-McKay-2013 Perou-Botstein-1999 Ramaswamy-Golub-2001 Rieger-Chu-2004 Roth-Zlotnik-2006 Salaverria-Siebert-2011 Smirnov-Cheung-2009 Wang-Maris-2006 Wu-Garvey-2007

Co-localization:
Johnson-Shoemaker-2003 Satoh-Yamamoto-2013 Schadt-Shoemaker-2004

Genetic interactions:
BIOGRID-SMALL-SCALE-STUDIES IREF-SMALL-SCALE-STUDIES Lin-Boeke-2012 A Lin-Boeke-2012 B Lin-Smith-2010 Toyoshima-Grandori-2012 Willingham-Muchowski-2003

Pathway:
PATHWAYCOMMONS-CELL_MAP PATHWAYCOMMONS-HUMANCYC PATHWAYCOMMONS-IMID PATHWAYCOMMONS-NCI_NATURE PATHWAYCOMMONS-REACTOME Wu-Stein-2010

Physical interactions:
Abu-Odeh-Aqeilan-2014 Agrawal-Sedivy-2010 Aichem-Groettrup-2012 Albers-Koegl-2005 Alexandru-Deshaies-2008 Altun-Kessler-2011 Andresen-Flores-Morales-2014 Arbuckle-Grant-2010 Arora-Mercola-2008 BIOGRID-SMALL-SCALE-STUDIES Bandyopadhyay-Ideker-2010 Bantscheff-Drewes-2011 Barr-Knapp-2009 Barrios-Rodiles-Wrana-2005 Behrends-Harper-2010 Behzadnia-Lührmann-2007 Bennett-Harper-2010 Benzinger-Hermeking-2005 Berggård-James-2006 Bett-Hay-2013 Blandin-Richard-2013 Bouwmeester-Superti-Furga-2004 Brajenovic-Drewes-2004 Brehme-Superti-Furga-2009 Bruderer-Hay-2011 Byron-Humphries-2012 Cai-Conaway-2007 Camargo-Brandon-2007 Cannavo-Jiricny-2007 Cao-Chinnaiyan-2014 Chen-Ge-2013 Chen-Naus-2012 Chen-Zhang-2013 Cheng-Chen-2010 Cloutier-Coulombe-2013 Colland-Gauthier-2004 Couzens-Gingras-2013 Cox-Rizzino-2013 Danielsen-Nielsen-2011 Dyer-Sobral-2010 Emanuele-Elledge-2011 Ewing-Figeys-2007 Fenner-Prehn-2010 Foerster-Ritter-2013 Foster-Marshall-2013 Freibaum-Taylor-2010 Gao-Reinberg-2012 Gautier-Hall-2009 Giannone-Liu-2010 Glatter-Gstaiger-2009 Gloeckner-Ueffing-2007 Goehler-Wanker-2004 Golebiowski-Hay-2009 Goudreault-Gingras-2009 Grant-2010 Greco-Cristea-2011 Havrylov-Redowicz-2009 Havugimana-Emili-2012 Hayes-Urbé-2012 Hegele-Stelzl-2012 A Hegele-Stelzl-2012 B Humphries-Humphries-2009 Hutchins-Peters-2010 IREF-BIND IREF-BIOGRID IREF-DIP IREF-HPRD IREF-INNATEDB IREF-INTACT IREF-MATRIXDB IREF-MPPI IREF-OPHID IREF-PUBMED

IREF-SMALL-SCALE-STUDIES Ingham-Pawson-2005 Jeronimo-Coulombe-2007 Jin-Pawson-2004 Jones-MacBeath-2006 Joshi-Cristea-2013 Jäger-Krogan-2012 Kahle-Zoghbi-2011 Kim-Gygi-2011 Kneissl-Grummt-2003 Koch-Hermeking-2007 Kristensen-Foster-2012 Lau-Ronai-2012 Lee-Doedens-2011 Lee-Songyang-2011 Lehner-Sanderson-2004 A Lehner-Sanderson-2004 B Leng-Wang-2014 Li-Dorf-2011 A Li-Dorf-2011 B Lim-Zoghbi-2006 Liu-Wang-2012 Loch-Strickler-2012 Lopitz-Otsoa-Rodriguez-2012 Lu-Zhang-2013 Mak-Moffat-2010 Malovannaya-Qin-2010 Markson-Sanderson-2009 Maréchal-Zou-2014 Matafora-Bachi-2009 Matsumoto-Nakayama-2005 McCracken-Blencowe-2005 McFarland-Nussbaum-2008 Meek-Piwnica-Worms-2004 Meierhofer-Kaiser-2008 Miyamoto-Sato-Yanagawa-2010 Nakayama-Ohara-2002 Nakayasu-Adkins-2013 Napolitano-Meroni-2011 Nathan-Goldberg-2013 Neganova-Lako-2011 Newman-Keating-2003 Olma-Pintard-2009 Oláh-Ovádi-2011 Oshikawa-Nakayama-2012 Ouyang-Gill-2009 Panigrahi-Pati-2012 Perez-Hernandez-Yáñez-Mó-2013 Persaud-Rotin-2009 Pichlmair-Superti-Furga-2011 Pichlmair-Superti-Furga-2012 Pilot-Storck-Goillot-2010 Povlsen-Choudhary-2012 Ramachandran-LaBaer-2004 Ravasi-Hayashizaki-2010 Reinke-Keating-2013 Richter-Chrzanowska-Lightowlers-2010 Roux-Burke-2012 Rowbotham-Mermoud-2011 Roy-Parent-2013 Rual-Vidal-2005 A Rual-Vidal-2005 B San-Marina-Minden-2008 Sang-Jackson-2011 Sato-Conaway-2004 Shi-Qin-2011 Singh-Moore-2012 Soler-López-Aloy-2011 Sowa-Harper-2009 Stehling-Lill-2012 Stelzl-Wanker-2005 Suter-Wanker-2013 Taipale-Lindquist-2012 Takahashi-Conaway-2011 Tarallo-Weisz-2011 Tatham-Hay-2011 Teixeira-Gomes-2010 Thalappilly-Dusetti-2008 Tsai-Cristea-2012 Udeshi-Carr-2012 Vandamme-Angrand-2011 Vanderwerf-Bagby-2009 Varjosalo-Gstaiger-2013 A Varjosalo-Gstaiger-2013 B Varjosalo-Superti-Furga-2013 Venkatesan-Vidal-2009 Vinayagam-Wanker-2011 Wagner-Choudhary-2011 Wallach-Kramer-2013 Wang-Balch-2006 Wang-He-2008 Wang-Yang-2011 Weimann-Stelzl-2013 A Weimann-Stelzl-2013 B Weinmann-Meister-2009 Wilker-Yaffe-2007 Wong-O'Bryan-2012 Woods-Monteiro-2012 Woodsmith-Sanderson-2012 Wu-Li-2007 Wu-Ma-2012 Xiao-Lefkowitz-2007 Xie-Cong-2013 Xu-Jaffrey-2010 Xu-Ye-2012 Yang-Chen-2010 Yatim-Benkirane-2012 Yu-Chow-2013 Yu-Vidal-2011 Zanon-Pichler-2013 Zhang-Zou-2011 Zhao-Krug-2005 Zhao-Yang-2011 Zhou-Conrads-2004 Zhou-Liang-2012 de Hoog-Mann-2004 van Wijk-Timmers-2009

Predicted:
I2D-BIND-Fly2Human I2D-BIND-Mouse2Human I2D-BIND-Rat2Human I2D-BIND-Worm2Human I2D-BIND-Yeast2Human I2D-BioGRID-Fly2Human I2D-BioGRID-Mouse2Human I2D-BioGRID-Rat2Human I2D-BioGRID-Worm2Human I2D-BioGRID-Yeast2Human I2D-Chen-Pawson-2009-PiwiScreen-Mouse2Human I2D-Formstecher-Daviet-2005-Embryo-Fly2Human I2D-Giot-Rothbert-2003-Low-Fly2Human I2D-INNATEDB-Mouse2Human I2D-IntAct-Fly2Human I2D-IntAct-Mouse2Human I2D-IntAct-Rat2Human I2D-IntAct-Worm2Human I2D-IntAct-Yeast2Human I2D-Krogan-Greenblatt-2006-Core-Yeast2Human I2D-Krogan-Greenblatt-2006-NonCore-Yeast2Human I2D-Li-Vidal-2004-CORE-1-Worm2Human I2D-Li-Vidal-2004-non-core-Worm2Human I2D-MGI-Mouse2Human I2D-MINT-Fly2Human I2D-MINT-Mouse2Human I2D-MINT-Rat2Human I2D-MINT-Worm2Human I2D-MINT-Yeast2Human I2D-Manual-Mouse2Human I2D-Manual-Rat2Human I2D-Ptacek-Snyder-2005-Yeast2Human I2D-Tarassov-PCA-Yeast2Human I2D-Tewari-Vidal-2004-TGFb-Worm2Human I2D-Wang-Orkin-2006-EScmplx-Mouse2Human I2D-Wang-Orkin-2006-EScmplxlow-Mouse2Human I2D-Yu-Vidal-2008-GoldStd-Yeast2Human I2D-vonMering-Bork-2002-High-Yeast2Human I2D-vonMering-Bork-2002-Low-Yeast2Human I2D-vonMering-Bork-2002-Medium-Yeast2Human Stuart-Kim-2003

Shared protein domains:
INTERPRO PFAM

Network weighting: Automatically selected weighting method (Biological process based)

Number of gene results: 20

Networks

Physical interactions **64.66 %**

Leng-Wang-2014 **< 0.01 %**

A proteomics strategy for the identification of FAT10-modified sites by mass spectrometry. Leng et al. (2014). *J Proteome Res*.

Source: Direct interaction with 140 interactions from iRefIndex

Zhao-Yang-2011 **< 0.01 %**

Interactome study suggests multiple cellular functions of hepatoma-derived growth factor (HDGF). Zhao et al. (2011). *J Proteomics*.

Source: Direct interaction with 161 interactions from iRefIndex

Soler-López-Aloy-2011 **< 0.01 %**

Interactome mapping suggests new mechanistic details underlying Alzheimer's disease. Soler-López et al. (2011). *Genome Res*.

Source: Direct interaction with 312 interactions from iRefIndex

Tags: fibroblasts; cultured cells; cell line; immune system

Takahashi-Conaway-2011 **< 0.01 %**

Human mediator subunit MED26 functions as a docking site for transcription elongation factors. Takahashi et al. (2011). *Cell*.

Source: Direct interaction with 131 interactions from iRefIndex

Tags: cell proliferation; transcription factors; cancer

Zhou-Liang-2012 **6.59 %**

Shotgun proteomics and network analysis of ubiquitin-related proteins from human breast carcinoma epithelial cells. Zhou et al. (2012). *Mol Cell Biochem*.

Source: Direct interaction with 207 interactions from BioGRID

Tags: cultured cells; cancer; epithelial cells; cell line; transcription factors; breast cancer

Singh-Moore-2012 **4.76 %**

The cellular EJC interactome reveals higher-order mRNP structure and an EJC-SR protein nexus. Singh et al. (2012). *Cell*.

Source: Direct interaction with 301 interactions from iRefIndex

Altun-Kessler-2011 **3.63 %**

Activity-based chemical proteomics accelerates inhibitor development for deubiquitylating enzymes. Altun et al. (2011). *Chem Biol*.

Source: Direct interaction with 287 interactions from BioGRID

Tags: cultured cells; cell line

Matsumoto-Nakayama-2005 **2.64 %**

Large-scale analysis of the human ubiquitin-related proteome. Matsumoto et al. (2005). *Proteomics*.

Source: Direct interaction with 648 interactions from BioGRID

Tags: cultured cells; cell line

Lee-Doedens-2011 **2.56 %**

Ubiquitin ligase substrate identification through quantitative proteomics at both the protein and peptide levels. Lee et al. (2011). *J Biol Chem*.

Source: Direct interaction with 1,320 interactions from BioGRID

Jones-MacBeath-2006 **1.88 %**

A quantitative protein interaction network for the ErbB receptors using protein microarrays. Jones et al. (2006). *Nature*.

Source: Direct interaction with 172 interactions from iRefIndex

Tags: cultured cells; cell line

Behzadnia-Lührmann-2007 **1.82 %**

Composition and three-dimensional EM structure of double affinity-purified, human prespliceosomal A complexes. Behzadnia et al. (2007). *EMBO J*.

Source: Direct interaction with 112 interactions from iRefIndex

Maréchal-Zou-2014 **1.65 %**

PRP19 transforms into a sensor of RPA-ssDNA after DNA damage and drives ATR activation via a ubiquitin-mediated circuitry. Maréchal et al. (2014). *Mol Cell*.

Source: Direct interaction with 996 interactions from BioGRID

Tags: transcription factors; signal transduction

Shi-Qin-2011 **1.60 %**

A data set of human endogenous protein ubiquitination sites. Shi et al. (2011). *Mol Cell Proteomics*.

Source: Direct interaction with 269 interactions from BioGRID

Tags: cancer

Berggård-James-2006 **1.60 %**

140 mouse brain proteins identified by Ca2+-calmodulin affinity chromatography and tandem mass spectrometry. Berggård et al. (2006). *J Proteome Res*.

Source: Direct interaction with 151 interactions from iRefIndex

Tags: brain; nervous system

Vanderwerf-Bagby-2009 **1.59 %**

TLR8-dependent TNF-(alpha) overexpression in Fanconi anemia group C cells. Vanderwerf et al. (2009). *Blood*.

Source: Direct interaction with 149 interactions from BioGRID

Tags: knockout; cell signalling; signal transduction

Mak-Moffat-2010 **1.42 %**

A lentiviral functional proteomics approach identifies chromatin remodeling complexes important for the induction of pluripotency. Mak et al. (2010). *Mol Cell Proteomics*.

Source: Direct interaction with 108 interactions from BioGRID

Tags: transcription factors; cell line; cultured cells; stem cells

McCracken-Blencowe-2005 **1.32 %**

Proteomic analysis of SRm160-containing complexes reveals a conserved association with cohesin. McCracken et al. (2005). *J Biol Chem*.

Source: Direct interaction with 177 interactions from iRefIndex

Neganova-Lako-2011 **1.31 %**

An important role for CDK2 in G1 to S checkpoint activation and DNA damage response in human embryonic stem cells. Neganova et al. (2011). *Stem Cells*.

Source: Direct interaction with 388 interactions from iRefIndex

Tags: development; cultured cells; cancer; cell proliferation; apoptosis; cell line; stem cells; transcription factors

McFarland-Nussbaum-2008 **1.01 %**

Proteomics analysis identifies phosphorylation-dependent alpha-synuclein protein interactions. McFarland et al. (2008). *Mol Cell Proteomics*.

Source: Direct interaction with 157 interactions from iRefIndex

Tags: brain; nervous system

Bett-Hay-2013 **0.93 %**

The P-body component USP52/PAN2 is a novel regulator of HIF1A mRNA stability. Bett et al. (2013). *Biochem J*.

Source: Direct interaction with 317 interactions from iRefIndex

Tags: transcription factors; cell line; cultured cells; cancer

Udeshi-Carr-2012 **0.93 %**

Methods for quantification of in vivo changes in protein ubiquitination following proteasome and deubiquitinase inhibition. Udeshi et al. (2012). *Mol Cell Proteomics*.

Source: Direct interaction with 2,481 interactions from BioGRID

Tags: cultured cells; cell line; cancer; immune system

Oshikawa-Nakayama-2012 **0.86 %**

Proteome-wide identification of ubiquitylation sites by conjugation of engineered lysine-less ubiquitin. Oshikawa et al. (2012). *J Proteome Res*.

Source: Direct interaction with 895 interactions from BioGRID

Gloeckner-Ueffing-2007 **0.80 %**

A novel tandem affinity purification strategy for the efficient isolation and characterisation of native protein complexes. Gloeckner et al. (2007). *Proteomics*.

Source: Direct interaction with 100 interactions from BioGRID

Tags: cell line; cultured cells; cancer

Jeronimo-Coulombe-2007 **0.78 %**

Systematic analysis of the protein interaction network for the human transcription machinery reveals the identity of the 7SK capping enzyme. Jeronimo et al. (2007). *Mol Cell*.

Source: Direct interaction with 699 interactions from BioGRID

Tags: cultured cells; cell line

Xu-Ye-2012 **0.76 %**

SGTA recognizes a noncanonical ubiquitin-like domain in the Bag6-Ubl4A-Trc35 complex to promote endoplasmic reticulum-associated degradation. Xu et al. (2012). *Cell Rep*.

Source: Direct interaction with 224 interactions from iRefIndex

Tags: cultured cells; cell line

Zhou-Conrads-2004 **0.74 %**

An investigation into the human serum "interactome". Zhou et al. (2004). *Electrophoresis*.

Source: Direct interaction with 160 interactions from iRefIndex

Yu-Chow-2013 **0.70 %**

VCP phosphorylation-dependent interaction partners prevent apoptosis in Helicobacter pylori-infected gastric epithelial cells. Yu et al. (2013). *PLoS One*.

Source: Direct interaction with 272 interactions from iRefIndex

Tags: cultured cells; signal transduction; cancer; cell proliferation; apoptosis; cell line; epithelial cells

Varjosalo-Gstaiger-2013 B **0.69 %**

The protein interaction landscape of the human CMGC kinase group. Varjosalo et al. (2013). *Cell Rep*.

Note: One of 2 datasets produced from this publication.

Source: Direct interaction with 306 interactions from BioGRID

Tags: cultured cells; cell line

Freibaum-Taylor-2010 **0.69 %**

Global analysis of TDP-43 interacting proteins reveals strong association with RNA splicing and translation machinery. Freibaum et al. (2010). *J Proteome Res*.

Source: Direct interaction with 222 interactions from BioGRID

Tags: transcription factors; cultured cells; cell line; immune system

Hegele-Stelzl-2012 B **0.68 %**

Dynamic protein-protein interaction wiring of the human spliceosome. Hegele et al. (2012). *Mol Cell*.

Note: One of 2 datasets produced from this publication.

Source: Direct interaction with 600 interactions from BioGRID

Brehme-Superti-Furga-2009 **0.67 %**

Charting the molecular network of the drug target Bcr-Abl. Brehme et al. (2009). *Proc Natl Acad Sci U S A*.

Source: Direct interaction with 579 interactions from iRefIndex

Tags: cultured cells; cancer; cell line

Weinmann-Meister-2009 **0.63 %**

Importin 8 is a gene silencing factor that targets argonaute proteins to distinct mRNAs. Weinmann et al. (2009). *Cell*.

Source: Direct interaction with 96 interactions from BioGRID

Tags: cultured cells; cell line

Agrawal-Sedivy-2010 **0.61 %**

Proteomic profiling of Myc-associated proteins. Agrawal et al. (2010). *Cell Cycle*.

Source: Direct interaction with 102 interactions from iRefIndex

Tags: transcription factors; cell line; cultured cells; cancer

Varjosalo-Superti-Furga-2013 **0.60 %**

Interlaboratory reproducibility of large-scale human protein-complex analysis by standardized AP-MS. Varjosalo et al. (2013). *Nat Methods*.

Source: Direct interaction with 4,194 interactions from BioGRID

Tags: cultured cells; cell line

Danielsen-Nielsen-2011 **0.59 %**

Mass spectrometric analysis of lysine ubiquitylation reveals promiscuity at site level. Danielsen et al. (2011). *Mol Cell Proteomics*.

Source: Direct interaction with 5,424 interactions from BioGRID

Tags: cultured cells; cell line

Kristensen-Foster-2012 **0.59 %**

A high-throughput approach for measuring temporal changes in the interactome. Kristensen et al. (2012). *Nat Methods*.

Source: Direct interaction with 7,104 interactions from BioGRID

Tags: time series

Cannavo-Jiricny-2007 **0.54 %**

Characterization of the interactome of the human MutL homologues MLH1, PMS1, and PMS2. Cannavo et al. (2007). *J Biol Chem*.

Source: Direct interaction with 101 interactions from iRefIndex

Tags: transcription factors; cell line; cultured cells; cancer

Rowbotham-Mermoud-2011 **0.53 %**

Maintenance of silent chromatin through replication requires SWI/SNF-like chromatin remodeler SMARCAD1. Rowbotham et al. (2011). *Mol Cell*.

Source: Direct interaction with 114 interactions from iRefIndex

Tags: cell proliferation; fibroblasts; cell line; cancer; cultured cells

Barr-Knapp-2009 **0.52 %**

Large-scale structural analysis of the classical human protein tyrosine phosphatome. Barr et al. (2009). *Cell*.

Source: Direct interaction with 165 interactions from iRefIndex

Foerster-Ritter-2013 **0.52 %**

Characterization of the EGFR interactome reveals associated protein complex networks and intracellular receptor dynamics. Foerster et al. (2013). *Proteomics*.

Source: Direct interaction with 161 interactions from iRefIndex

IREF-DIP **0.51 %**

Source: Direct interaction with 3,719 interactions from iRefIndex

Cox-Rizzino-2013 **0.49 %**

The SOX2-interactome in brain cancer cells identifies the requirement of MSI2 and USP9X for the growth of brain tumor cells. Cox et al. (2013). *PLoS One*.

Source: Direct interaction with 280 interactions from iRefIndex

Tags: cell proliferation; transcription factors; cell line; cultured cells; cancer

Havugimana-Emili-2012 **0.49 %**

A census of human soluble protein complexes. Havugimana et al. (2012). *Cell*.

Source: Direct interaction with 13,710 interactions from BioGRID

Giannone-Liu-2010 **0.46 %**

The protein network surrounding the human telomere repeat binding factors TRF1, TRF2, and POT1. Giannone et al. (2010). *PLoS One*.

Source: Direct interaction with 279 interactions from iRefIndex

Tags: transcription factors; cultured cells; cell line

Li-Dorf-2011 A **0.46 %**

Mapping a dynamic innate immunity protein interaction network regulating type I interferon production. Li et al. (2011). *Immunity*.

Note: One of 2 datasets produced from this publication.

Source: Direct interaction with 400 interactions from BioGRID

Tags: cell signalling; cultured cells; cell line; immune system

Persaud-Rotin-2009 **0.44 %**

Comparison of substrate specificity of the ubiquitin ligases Nedd4 and Nedd4-2 using proteome arrays. Persaud et al. (2009). *Mol Syst Biol*.

Source: Direct interaction with 244 interactions from iRefIndex

IREF-MATRIXDB **0.44 %**

Source: Direct interaction with 179 interactions from iRefIndex

Wagner-Choudhary-2011 **0.41 %**

A proteome-wide, quantitative survey of in vivo ubiquitylation sites reveals widespread regulatory roles. Wagner et al. (2011). *Mol Cell Proteomics*.

Source: Direct interaction with 4,590 interactions from BioGRID

Tags: signal transduction

Varjosalo-Gstaiger-2013 A **0.39 %**

The protein interaction landscape of the human CMGC kinase group. Varjosalo et al. (2013). *Cell Rep*.

Note: One of 2 datasets produced from this publication.

Source: Direct interaction with 686 interactions from BioGRID

Tags: cultured cells; cell line

Roux-Burke-2012 **0.39 %**

A promiscuous biotin ligase fusion protein identifies proximal and interacting proteins in mammalian cells. Roux et al. (2012). *J Cell Biol*.

Source: Direct interaction with 113 interactions from iRefIndex

Tags: transcription factors

BIOGRID-SMALL-SCALE-STUDIES **0.37 %**

Source: Direct interaction with 51,715 interactions from BioGRID

Zhao-Krug-2005 **0.35 %**

Human ISG15 conjugation targets both IFN-induced and constitutively expressed proteins functioning in diverse cellular pathways. Zhao et al. (2005). *Proc Natl Acad Sci U S A*.

Source: Direct interaction with 149 interactions from iRefIndex

Tags: cell signalling; immune system

Hutchins-Peters-2010 **0.35 %**

Systematic analysis of human protein complexes identifies chromosome segregation proteins. Hutchins et al. (2010). *Science*.

Source: Direct interaction with 1,770 interactions from BioGRID

Tags: cell proliferation; nervous system; localization

Loch-Strickler-2012 **0.33 %**

A microarray of ubiquitylated proteins for profiling deubiquitylase activity reveals the critical roles of both chain and substrate. Loch et al. (2012). *Biochim Biophys Acta*.

Source: Direct interaction with 147 interactions from iRefIndex

Sowa-Harper-2009 **0.33 %**

Defining the human deubiquitinating enzyme interaction landscape. Sowa et al. (2009). *Cell*.

Source: Direct interaction with 1,506 interactions from BioGRID

Roy-Parent-2013 **0.33 %**

Novel, gel-free proteomics approach identifies RNF5 and JAMP as modulators of GPCR stability. Roy et al. (2013). *Mol Endocrinol*.

Source: Direct interaction with 153 interactions from iRefIndex

Tags: transcription factors; signal transduction; cell line; cultured cells

IREF-INNATEDB **0.33 %**

Source: Direct interaction with 3,870 interactions from iRefIndex

Taipale-Lindquist-2012 **0.30 %**

Quantitative analysis of HSP90-client interactions reveals principles of substrate recognition. Taipale et al. (2012). *Cell*.

Source: Direct interaction with 726 interactions from iRefIndex

Tags: transcription factors

Barrios-Rodiles-Wrana-2005 **0.28 %**

High-throughput mapping of a dynamic signaling network in mammalian cells. Barrios-Rodiles et al. (2005). *Science*.

Source: Direct interaction with 611 interactions from iRefIndex

Tags: cell signalling; cell line; cancer; epithelial cells; signal transduction; cultured cells; transcription factors

Wilker-Yaffe-2007 **0.28 %**

14-3-3sigma controls mitotic translation to facilitate cytokinesis. Wilker et al. (2007). *Nature*.

Source: Direct interaction with 110 interactions from iRefIndex

Tags: cell proliferation; transcription factors; cell line; cultured cells; cancer

IREF-MPPI **0.27 %**

Source: Direct interaction with 384 interactions from iRefIndex

Malovannaya-Qin-2010 **0.27 %**

Streamlined analysis schema for high-throughput identification of endogenous protein complexes. Malovannaya et al. (2010). *Proc Natl Acad Sci U S A*.

Source: Direct interaction with 235 interactions from iRefIndex

Jin-Pawson-2004 **0.26 %**

Proteomic, functional, and domain-based analysis of in vivo 14-3-3 binding proteins involved in cytoskeletal regulation and cellular organization. Jin et al. (2004). *Curr Biol*.

Source: Direct interaction with 233 interactions from iRefIndex

Tags: cell proliferation; cultured cells; immune system

Woods-Monteiro-2012 **0.25 %**

Charting the landscape of tandem BRCT domain-mediated protein interactions. Woods et al. (2012). *Sci Signal*.

Source: Direct interaction with 927 interactions from iRefIndex

Tags: transcription factors; cancer

Fenner-Prehn-2010 **0.24 %**

Expanding the substantial interactome of NEMO using protein microarrays. Fenner et al. (2010). *PLoS One*.

Source: Direct interaction with 106 interactions from iRefIndex

Tags: transcription factors; cultured cells; signal transduction; cell line

Xiao-Lefkowitz-2007 **0.23 %**

Functional specialization of beta-arrestin interactions revealed by proteomic analysis. Xiao et al. (2007). *Proc Natl Acad Sci U S A*.

Source: Direct interaction with 402 interactions from iRefIndex

Tags: signal transduction

IREF-PUBMED **0.23 %**

Source: Direct interaction with 459 interactions from iRefIndex

Xie-Cong-2013 **0.23 %**

Deubiquitinase FAM/USP9X interacts with the E3 ubiquitin ligase SMURF1 protein and protects it from ligase activity-dependent self-degradation. Xie et al. (2013). *J Biol Chem*.

Source: Direct interaction with 168 interactions from iRefIndex

Tags: cell line; cultured cells; cancer

IREF-BIND **0.22 %**

Source: Direct interaction with 7,078 interactions from iRefIndex

Koch-Hermeking-2007 **0.22 %**

Large-scale identification of c-MYC-associated proteins using a combined TAP/MudPIT approach. Koch et al. (2007). *Cell Cycle*.

Source: Direct interaction with 179 interactions from iRefIndex

Tags: transcription factors; cell line; cultured cells; cancer

Chen-Zhang-2013 **0.21 %**

Quantitative study of the interactome of PKC? involved in the EGF-induced tumor cell chemotaxis. Chen et al. (2013). *J Proteome Res*.

Source: Direct interaction with 181 interactions from iRefIndex

Tags: cell line; cultured cells; breast cancer; cancer

Napolitano-Meroni-2011 **0.19 %**

Functional interactions between ubiquitin E2 enzymes and TRIM proteins. Napolitano et al. (2011). *Biochem J*.

Source: Direct interaction with 81 interactions from BioGRID

Tags: transcription factors; cultured cells; nervous system

Lee-Songyang-2011 **0.18 %**

Genome-wide YFP fluorescence complementation screen identifies new regulators for telomere signaling in human cells. Lee et al. (2011). *Mol Cell Proteomics*.

Source: Direct interaction with 603 interactions from iRefIndex

Tags: transcription factors; signal transduction

Brajenovic-Drewes-2004 **0.17 %**

Comprehensive proteomic analysis of human Par protein complexes reveals an interconnected protein network. Brajenovic et al. (2004). *J Biol Chem*.

Source: Direct interaction with 125 interactions from iRefIndex

Tags: cultured cells; cell line

Yatim-Benkirane-2012 **0.17 %**

NOTCH1 nuclear interactome reveals key regulators of its transcriptional activity and oncogenic function. Yatim et al. (2012). *Mol Cell*.

Source: Direct interaction with 131 interactions from iRefIndex

Tags: transcription factors; cell line; lymphoma; cancer; cultured cells

Bandyopadhyay-Ideker-2010 **0.16 %**

A human MAP kinase interactome. Bandyopadhyay et al. (2010). *Nat Methods*.

Source: Direct interaction with 625 interactions from iRefIndex

Tags: transcription factors; signal transduction; cell line; cultured cells

Bantscheff-Drewes-2011 **0.16 %**

Chemoproteomics profiling of HDAC inhibitors reveals selective targeting of HDAC complexes. Bantscheff et al. (2011). *Nat Biotechnol*.

Source: Direct interaction with 103 interactions from BioGRID

Zanon-Pichler-2013 **0.16 %**

Profiling of Parkin-binding partners using tandem affinity purification. Zanon et al. (2013). *PLoS One*.

Source: Direct interaction with 194 interactions from BioGRID

Wu-Li-2007 **0.16 %**

Systematic identification of SH3 domain-mediated human protein-protein interactions by peptide array target screening. Wu et al. (2007). *Proteomics*.

Source: Direct interaction with 923 interactions from iRefIndex

Tags: cultured cells; signal transduction; cell line

Liu-Wang-2012 **0.15 %**

Proteomic identification of common SCF ubiquitin ligase FBXO6-interacting glycoproteins in three kinds of cells. Liu et al. (2012). *J Proteome Res*.

Source: Direct interaction with 594 interactions from BioGRID

Tags: cell line; cultured cells; cancer; immune system

Ewing-Figeys-2007 **0.15 %**

Large-scale mapping of human protein-protein interactions by mass spectrometry. Ewing et al. (2007). *Mol Syst Biol*.

Source: Direct interaction with 5,673 interactions from iRefIndex

Oláh-Ovádi-2011 **0.15 %**

Interactions of pathological hallmark proteins: tubulin polymerization promoting protein/p25, beta-amyloid, and alpha-synuclein. Oláh et al. (2011). *J Biol Chem*.

Source: Direct interaction with 1,851 interactions from iRefIndex

Tags: epithelial cells; cultured cells; nervous system; cell line

Tsai-Cristea-2012 **0.15 %**

Functional proteomics establishes the interaction of SIRT7 with chromatin remodeling complexes and expands its role in regulation of RNA polymerase I transcription. Tsai et al. (2012). *Mol Cell Proteomics*.

Source: Direct interaction with 654 interactions from iRefIndex

Tags: transcription factors; cell line; cultured cells; localization

Chen-Naus-2012 **0.15 %**

Association of connexin43 with E3 ubiquitin ligase TRIM21 reveals a mechanism for gap junction phosphodegron control. Chen et al. (2012). *J Proteome Res*.

Source: Direct interaction with 106 interactions from iRefIndex

Tags: cancer

Ingham-Pawson-2005 **0.14 %**

WW domains provide a platform for the assembly of multiprotein networks. Ingham et al. (2005). *Mol Cell Biol*.

Source: Direct interaction with 297 interactions from iRefIndex

Tags: cultured cells; cell line; cancer; immune system

van Wijk-Timmers-2009 **0.14 %**

A comprehensive framework of E2-RING E3 interactions of the human ubiquitin-proteasome system. van Wijk et al. (2009). *Mol Syst Biol*.

Source: Direct interaction with 306 interactions from iRefIndex

Tags: cell line; cultured cells; cancer

Behrends-Harper-2010 **0.12 %**

Network organization of the human autophagy system. Behrends et al. (2010). *Nature*.

Source: Direct interaction with 766 interactions from iRefIndex

Hayes-Urbé-2012 **0.11 %**

Direct and indirect control of mitogen-activated protein kinase pathway-associated components, BRAP/IMP E3 ubiquitin ligase and CRAF/RAF1 kinase, by the deubiquitylating enzyme USP15. Hayes et al. (2012). *J Biol Chem*.

Source: Direct interaction with 107 interactions from iRefIndex

Tags: cultured cells; signal transduction; cancer; cell line

Couzens-Gingras-2013 **0.11 %**

Protein interaction network of the mammalian Hippo pathway reveals mechanisms of kinase-phosphatase interactions. Couzens et al. (2013). *Sci Signal*.

Source: Direct interaction with 366 interactions from BioGRID

IREF-INTACT **0.11 %**

Source: Direct interaction with 42,475 interactions from iRefIndex

Kim-Gygi-2011 **0.11 %**

Systematic and quantitative assessment of the ubiquitin-modified proteome. Kim et al. (2011). *Mol Cell*.

Source: Direct interaction with 4,905 interactions from BioGRID

Tags: cell line; cultured cells; cancer

Arbuckle-Grant-2010 **0.11 %**

The SH3 domain of postsynaptic density 95 mediates inflammatory pain through phosphatidylinositol-3-kinase recruitment. Arbuckle et al. (2010). *EMBO Rep*.

Source: Direct interaction with 268 interactions from iRefIndex

Tags: brain; immune system

Greco-Cristea-2011 **0.10 %**

Nuclear import of histone deacetylase 5 by requisite nuclear localization signal phosphorylation. Greco et al. (2011). *Mol Cell Proteomics*.

Source: Direct interaction with 240 interactions from iRefIndex

Tags: cultured cells; cell line; localization

Joshi-Cristea-2013 **0.10 %**

The functional interactome landscape of the human histone deacetylase family. Joshi et al. (2013). *Mol Syst Biol*.

Source: Direct interaction with 307 interactions from iRefIndex

Tags: cell proliferation; transcription factors; signal transduction; immune system

Bennett-Harper-2010 **0.10 %**

Dynamics of cullin-RING ubiquitin ligase network revealed by systematic quantitative proteomics. Bennett et al. (2010). *Cell*.

Source: Direct interaction with 4,386 interactions from BioGRID

Tags: transcription factors; cultured cells; cell line; hormones

Cai-Conaway-2007 **0.09 %**

YY1 functions with INO80 to activate transcription. Cai et al. (2007). *Nat Struct Mol Biol*.

Source: Direct interaction with 106 interactions from BioGRID

Tags: transcription factors; cultured cells; cell line

Thalappilly-Dusetti-2008 **0.08 %**

Identification of multi-SH3 domain-containing protein interactome in pancreatic cancer: a yeast two-hybrid approach. Thalappilly et al. (2008). *Proteomics*.

Source: Direct interaction with 105 interactions from iRefIndex

Tags: transcription factors; cell line; cultured cells; cancer

IREF-HPRD **0.08 %**

Source: Direct interaction with 34,112 interactions from iRefIndex

Ouyang-Gill-2009 **0.07 %**

Direct binding of CoREST1 to SUMO-2/3 contributes to gene-specific repression by the LSD1/CoREST1/HDAC complex. Ouyang et al. (2009). *Mol Cell*.

Source: Direct interaction with 105 interactions from BioGRID

Tags: transcription factors; nervous system; cancer; cell line; cultured cells

Woodsmith-Sanderson-2012 **0.07 %**

Systematic analysis of dimeric E3-RING interactions reveals increased combinatorial complexity in human ubiquitination networks. Woodsmith et al. (2012). *Mol Cell Proteomics*.

Source: Direct interaction with 208 interactions from iRefIndex

IREF-OPHID **0.07 %**

Source: Direct interaction with 44,492 interactions from iRefIndex

Perez-Hernandez-Yáñez-Mó-2013 **0.07 %**

The intracellular interactome of tetraspanin-enriched microdomains reveals their function as sorting machineries toward exosomes. Perez-Hernandez et al. (2013). *J Biol Chem*.

Source: Direct interaction with 452 interactions from iRefIndex

Tags: knockout; cultured cells; cell line

Zhang-Zou-2011 **0.07 %**

A bead-based approach for large-scale identification of in vitro kinase substrates. Zhang et al. (2011). *Proteomics*.

Source: Direct interaction with 163 interactions from iRefIndex

Grant-2010 **0.07 %**

Identification of SUMOylated proteins in neuroblastoma cells after treatment with hydrogen peroxide or ascorbate. et al. (2010). *BMB Rep*.

Source: Direct interaction with 145 interactions from iRefIndex

Tags: cell line; cultured cells; cancer

Nakayasu-Adkins-2013 **0.06 %**

Evaluation of selected binding domains for the analysis of ubiquitinated proteomes. Nakayasu et al. (2013). *J Am Soc Mass Spectrom*.

Source: Direct interaction with 881 interactions from iRefIndex

Tags: cultured cells; signal transduction; cell line; immune system

Glatter-Gstaiger-2009 **0.06 %**

An integrated workflow for charting the human interaction proteome: insights into the PP2A system. Glatter et al. (2009). *Mol Syst Biol*.

Source: Direct interaction with 244 interactions from BioGRID

Tags: cultured cells; cell line

Ravasi-Hayashizaki-2010 **0.05 %**

An atlas of combinatorial transcriptional regulation in mouse and man. Ravasi et al. (2010). *Cell*.

Source: Direct interaction with 635 interactions from iRefIndex

Tags: cell proliferation; transcription factors

Golebiowski-Hay-2009 **0.04 %**

System-wide changes to SUMO modifications in response to heat shock. Golebiowski et al. (2009). *Sci Signal*.

Source: Direct interaction with 731 interactions from BioGRID

Tags: cell proliferation

Cheng-Chen-2010 **0.04 %**

Quantitative nanoproteomics for protein complexes (QNanoPX) related to estrogen transcriptional action. Cheng et al. (2010). *Mol Cell Proteomics*.

Source: Direct interaction with 259 interactions from iRefIndex

Tags: transcription factors; cell line; cancer; cultured cells

Lau-Ronai-2012 **0.04 %**

PKC? promotes oncogenic functions of ATF2 in the nucleus while blocking its apoptotic function at mitochondria. Lau et al. (2012). *Cell*.

Source: Direct interaction with 134 interactions from iRefIndex

Tags: cultured cells; cancer; apoptosis; cell line; fibroblasts; stem cells; transcription factors

Richter-Chrzanowska-Lightowlers-2010 **0.03 %**

A functional peptidyl-tRNA hydrolase, ICT1, has been recruited into the human mitochondrial ribosome. Richter et al. (2010). *EMBO J*.

Source: Direct interaction with 192 interactions from iRefIndex

Vinayagam-Wanker-2011 **0.03 %**

A directed protein interaction network for investigating intracellular signal transduction. Vinayagam et al. (2011). *Sci Signal*.

Source: Direct interaction with 2,526 interactions from iRefIndex

Tags: transcription factors; signal transduction

Yang-Chen-2010 **0.03 %**

Proteomic dissection of cell type-specific H2AX-interacting protein complex associated with hepatocellular carcinoma. Yang et al. (2010). *J Proteome Res*.

Source: Direct interaction with 100 interactions from BioGRID

Tags: cultured cells; cell line; signal transduction; cancer

Wong-O'Bryan-2012 **0.03 %**

Intersectin (ITSN) family of scaffolds function as molecular hubs in protein interaction networks. Wong et al. (2012). *PLoS One*.

Source: Direct interaction with 115 interactions from iRefIndex

Tags: fibroblasts; cultured cells; signal transduction; cell line

Markson-Sanderson-2009 **0.02 %**

Analysis of the human E2 ubiquitin conjugating enzyme protein interaction network. Markson et al. (2009). *Genome Res*.

Source: Direct interaction with 700 interactions from iRefIndex

Tags: cell line; cultured cells; cancer

Weimann-Stelzl-2013 A **0.02 %**

A Y2H-seq approach defines the human protein methyltransferase interactome. Weimann et al. (2013). *Nat Methods*.

Note: One of 2 datasets produced from this publication.

Source: Direct interaction with 114 interactions from BioGRID

Wallach-Kramer-2013 **0.02 %**

Dynamic circadian protein-protein interaction networks predict temporal organization of cellular functions. Wallach et al. (2013). *PLoS Genet*.

Source: Direct interaction with 110 interactions from iRefIndex

Tags: cell proliferation; transcription factors; signal transduction; biological clocks

Pichlmair-Superti-Furga-2012 **0.02 %**

Viral immune modulators perturb the human molecular network by common and unique strategies. Pichlmair et al. (2012). *Nature*.

Source: Direct interaction with 14 interactions from BioGRID

Tags: signal transduction; immune system

Meek-Piwnica-Worms-2004 **0.01 %**

Comprehensive proteomic analysis of interphase and mitotic 14-3-3-binding proteins. Meek et al. (2004). *J Biol Chem*.

Source: Direct interaction with 351 interactions from iRefIndex

Tags: cell proliferation

Co-expression **17.38 %**

Wang-Maris-2006 **1.23 %**

Integrative genomics identifies distinct molecular classes of neuroblastoma and shows that multiple genes are targeted by regional alterations in DNA copy number. Wang et al. (2006). *Cancer Res*.

Source: Pearson correlation with 264,234 interactions from GEO

Tags: transcription factors; cancer

Ramaswamy-Golub-2001 **1.16 %**

Multiclass cancer diagnosis using tumor gene expression signatures. Ramaswamy et al. (2001). *Proc Natl Acad Sci U S A*.

Source: Pearson correlation with 270,142 interactions from supplementary material

Tags: cancer

Bild-Nevins-2006 B **1.11 %**

Oncogenic pathway signatures in human cancers as a guide to targeted therapies. Bild et al. (2006). *Nature*.

Note: One of 3 datasets produced from this publication.

Source: Pearson correlation with 282,582 interactions from GEO

Tags: cultured cells; signal transduction; cancer; epithelial cells; cell line; disease; breast; transcription factors; breast cancer

Salaverria-Siebert-2011 **1.10 %**

Translocations activating IRF4 identify a subtype of germinal center-derived B-cell lymphoma affecting predominantly children and young adults. Salaverria et al. (2011). *Blood*.

Source: Pearson correlation with 514,070 interactions from GEO

Tags: transcription factors; cancer

Burington-Shaughnessy-2008 **1.00 %**

Tumor cell gene expression changes following short-term in vivo exposure to single agent chemotherapeutics are related to survival in multiple myeloma. Burington et al. (2008). *Clin Cancer Res*.

Source: Pearson correlation with 293,587 interactions from GEO

Tags: transcription factors; time series; cancer; chemotherapy

Bahr-Bowler-2013 **1.00 %**

Peripheral blood mononuclear cell gene expression in chronic obstructive pulmonary disease. Bahr et al. (2013). *Am J Respir Cell Mol Biol*.

Source: Pearson correlation with 278,447 interactions from GEO

Mallon-McKay-2013 **1.00 %**

StemCellDB: the human pluripotent stem cell database at the National Institutes of Health. Mallon et al. (2013). *Stem Cell Res*.

Source: Pearson correlation with 567,140 interactions from GEO

Tags: cultured cells; stem cells; cell line

Alizadeh-Staudt-2000 **0.96 %**

Distinct types of diffuse large B-cell lymphoma identified by gene expression profiling. Alizadeh et al. (2000). *Nature*.

Source: Pearson correlation with 88,888 interactions from supplementary material

Tags: cultured cells; cancer

Rieger-Chu-2004 **0.86 %**

Toxicity from radiation therapy associated with abnormal transcriptional responses to DNA damage. Rieger et al. (2004). *Proc Natl Acad Sci U S A*.

Source: Pearson correlation with 259,055 interactions from GEO

Tags: cultured cells; cell line

Smirnov-Cheung-2009 **0.82 %**

Genetic analysis of radiation-induced changes in human gene expression. Smirnov et al. (2009). *Nature*.

Source: Pearson correlation with 463,390 interactions from GEO

Tags: transcription factors; cultured cells; cell line

Roth-Zlotnik-2006 **0.81 %**

Gene expression analyses reveal molecular relationships among 20 regions of the human CNS. Roth et al. (2006). *Neurogenetics*.

Source: Pearson correlation with 666,614 interactions from GEO

Gysin-McMahon-2012 **0.80 %**

Analysis of mRNA profiles after MEK1/2 inhibition in human pancreatic cancer cell lines reveals pathways involved in drug sensitivity. Gysin et al. (2012). *Mol Cancer Res*.

Source: Pearson correlation with 388,454 interactions from GEO

Tags: cell proliferation; cultured cells; cell line; signal transduction; cancer

Innocenti-Brown-2011 **0.80 %**

Identification, replication, and functional fine-mapping of expression quantitative trait loci in primary human liver tissue. Innocenti et al. (2011). *PLoS Genet*.

Source: Pearson correlation with 579,361 interactions from GEO

Tags: epithelial cells; cultured cells; cell line; liver; cancer

Kang-Willman-2010 **0.77 %**

Gene expression classifiers for relapse-free survival and minimal residual disease improve risk classification and outcome prediction in pediatric B-precursor acute lymphoblastic leukemia. Kang et al. (2010). *Blood*.

Source: Pearson correlation with 656,632 interactions from GEO

Tags: transcription factors; lymphoma; cancer

Boldrick-Relman-2002 **0.74 %**

Stereotyped and specific gene expression programs in human innate immune responses to bacteria. Boldrick et al. (2002). *Proc Natl Acad Sci U S A*.

Source: Pearson correlation with 108,543 interactions from supplementary material

Tags: immune system

Arijs-Rutgeerts-2009 **0.73 %**

Mucosal gene expression of antimicrobial peptides in inflammatory bowel disease before and after first infliximab treatment. Arijs et al. (2009). *PLoS One*.

Source: Pearson correlation with 653,194 interactions from GEO

Tags: immune system

Wu-Garvey-2007 **0.64 %**

The effect of insulin on expression of genes and biochemical pathways in human skeletal muscle. Wu et al. (2007). *Endocrine*.

Source: Pearson correlation with 260,762 interactions from GEO

Tags: transcription factors; muscle; cultured cells

Chen-Brown-2002 **0.62 %**

Gene expression patterns in human liver cancers. Chen et al. (2002). *Mol Biol Cell*.

Source: Pearson correlation with 275,649 interactions from supplementary material

Tags: cell proliferation; transcription factors; liver; cancer

Cheok-Evans-2003 **0.61 %**

Treatment-specific changes in gene expression discriminate in vivo drug response in human leukemia cells. Cheok et al. (2003). *Nat Genet*.

Source: Pearson correlation with 263,940 interactions from GEO

Tags: chemotherapy; cultured cells; lymphoma; cancer

Perou-Botstein-1999 **0.61 %**

Distinctive gene expression patterns in human mammary epithelial cells and breast cancers. Perou et al. (1999). *Proc Natl Acad Sci U S A*.

Source: Pearson correlation with 62,886 interactions from supplementary material

Tags: cultured cells; cancer; epithelial cells; signal transduction; breast; stromal cells; transcription factors; breast cancer

Predicted **7.17 %**

I2D-Wang-Orkin-2006-EScmplx-Mouse2Human **< 0.01 %**

A protein interaction network for pluripotency of embryonic stem cells. Wang et al. (2006). *Nature*.

Note: I2D predictions of protein protein interactions using Wang-Orkin-2006 Mus musculus data

Source: Direct interaction with 53 interactions from I2D

Tags: development; transcription factors; stem cells

I2D-vonMering-Bork-2002-High-Yeast2Human **1.06 %**

Comparative assessment of large-scale data sets of protein-protein interactions. von Mering et al. (2002). *Nature*.

Note: I2D predictions of protein protein interactions using vonMering-Bork-2002 Saccharomyces cerevisiae data

Source: Direct interaction with 1,184 interactions from I2D

I2D-vonMering-Bork-2002-Medium-Yeast2Human **0.69 %**

Comparative assessment of large-scale data sets of protein-protein interactions. von Mering et al. (2002). *Nature*.

Note: I2D predictions of protein protein interactions using vonMering-Bork-2002 Saccharomyces cerevisiae data

Source: Direct interaction with 3,001 interactions from I2D

I2D-BioGRID-Yeast2Human **0.60 %**

BioGRID: a general repository for interaction datasets. Stark et al. (2006). *Nucleic Acids Res*.

Note: I2D predictions of protein protein interactions using BioGRID Saccharomyces cerevisiae data

Source: Direct interaction with 13,302 interactions from I2D

I2D-Chen-Pawson-2009-PiwiScreen-Mouse2Human **0.54 %**

Mouse Piwi interactome identifies binding mechanism of Tdrkh Tudor domain to arginine methylated Miwi. Chen et al. (2009). *Proc Natl Acad Sci U S A*.

Note: I2D predictions of protein protein interactions using Chen-Pawson-2009 Mus musculus data

Source: Direct interaction with 31 interactions from I2D

I2D-INNATEDB-Mouse2Human **0.47 %**

InnateDB: facilitating systems-level analyses of the mammalian innate immune response. Lynn et al. (2008). *Mol Syst Biol*.

Note: I2D predictions of protein protein interactions using INNATEDB Mus musculus data

Source: Direct interaction with 1,454 interactions from I2D

Tags: signal transduction; immune system

I2D-Tarassov-PCA-Yeast2Human **0.47 %**

An in vivo map of the yeast protein interactome. Tarassov et al. (2008). *Science*.

Note: I2D predictions of protein protein interactions using Tarassov-Michnick-2008 Saccharomyces cerevisiae data

Source: Direct interaction with 441 interactions from I2D

Tags: cell proliferation

I2D-vonMering-Bork-2002-Low-Yeast2Human **0.42 %**

Comparative assessment of large-scale data sets of protein-protein interactions. von Mering et al. (2002). *Nature*.

Note: I2D predictions of protein protein interactions using vonMering-Bork-2002 Saccharomyces cerevisiae data

Source: Direct interaction with 15,990 interactions from I2D

I2D-IntAct-Mouse2Human **0.35 %**

The IntAct molecular interaction database in 2010. Aranda et al. (2010). *Nucleic Acids Res*.

Note: I2D predictions of protein protein interactions using IntAct Mus musculus data

Source: Direct interaction with 3,430 interactions from I2D

Stuart-Kim-2003 **0.31 %**

A gene-coexpression network for global discovery of conserved genetic modules. Stuart et al. (2003). *Science*.

Source: 24,928 interactions from supplementary material

Tags: cell proliferation; cultured cells; signal transduction; cancer

I2D-Yu-Vidal-2008-GoldStd-Yeast2Human **0.28 %**

High-quality binary protein interaction map of the yeast interactome network. Yu et al. (2008). *Science*.

Note: I2D predictions of protein protein interactions using Yu Gold Standard Saccharomyces cerevisiae data

Source: Direct interaction with 376 interactions from I2D

Tags: transcription factors; signal transduction

I2D-Krogan-Greenblatt-2006-Core-Yeast2Human **0.25 %**

Global landscape of protein complexes in the yeast Saccharomyces cerevisiae. Krogan et al. (2006). *Nature*.

Note: I2D predictions of protein protein interactions using Krogan-Greenblatt-2006 Saccharomyces cerevisiae data

Source: Direct interaction with 1,822 interactions from I2D

I2D-BIND-Rat2Human **0.21 %**

BIND--a data specification for storing and describing biomolecular interactions, molecular complexes and pathways. Bader et al. (2000). *Bioinformatics*.

Note: I2D predictions of protein protein interactions using BIND Rattus norvegicus data

Source: Direct interaction with 548 interactions from I2D

I2D-MINT-Rat2Human **0.21 %**

MINT: a Molecular INTeraction database. Zanzoni et al. (2002). *FEBS Lett*.

Note: I2D predictions of protein protein interactions using MINT Rattus norvegicus data

Source: Direct interaction with 569 interactions from I2D

I2D-BioGRID-Worm2Human **0.19 %**

BioGRID: a general repository for interaction datasets. Stark et al. (2006). *Nucleic Acids Res*.

Note: I2D predictions of protein protein interactions using BioGRID Caenorhabditis elegans data

Source: Direct interaction with 944 interactions from I2D

I2D-MINT-Mouse2Human **0.19 %**

MINT: a Molecular INTeraction database. Zanzoni et al. (2002). *FEBS Lett*.

Note: I2D predictions of protein protein interactions using MINT Mus musculus data

Source: Direct interaction with 977 interactions from I2D

I2D-BIND-Mouse2Human **0.18 %**

BIND--a data specification for storing and describing biomolecular interactions, molecular complexes and pathways. Bader et al. (2000). *Bioinformatics*.

Note: I2D predictions of protein protein interactions using BIND Mus musculus data

Source: Direct interaction with 1,198 interactions from I2D

I2D-BioGRID-Mouse2Human **0.14 %**

BioGRID: a general repository for interaction datasets. Stark et al. (2006). *Nucleic Acids Res*.

Note: I2D predictions of protein protein interactions using BioGRID Mus musculus data

Source: Direct interaction with 284 interactions from I2D

I2D-BIND-Yeast2Human **0.13 %**

BIND--a data specification for storing and describing biomolecular interactions, molecular complexes and pathways. Bader et al. (2000). *Bioinformatics*.

Note: I2D predictions of protein protein interactions using BIND Saccharomyces cerevisiae data

Source: Direct interaction with 1,526 interactions from I2D

I2D-Krogan-Greenblatt-2006-NonCore-Yeast2Human **0.11 %**

Global landscape of protein complexes in the yeast Saccharomyces cerevisiae. Krogan et al. (2006). *Nature*.

Note: I2D predictions of protein protein interactions using Krogan-Greenblatt-2006 Saccharomyces cerevisiae data

Source: Direct interaction with 1,779 interactions from I2D

I2D-IntAct-Fly2Human **0.11 %**

The IntAct molecular interaction database in 2010. Aranda et al. (2010). *Nucleic Acids Res*.

Note: I2D predictions of protein protein interactions using IntAct Drosophila melanogaster data

Source: Direct interaction with 3,934 interactions from I2D

I2D-Formstecher-Daviet-2005-Embryo-Fly2Human **0.10 %**

Protein interaction mapping: a Drosophila case study. Formstecher et al. (2005). *Genome Res*.

Note: I2D predictions of protein protein interactions using Formstecher-Daviet-2005 Drosophila melanogaster data

Source: Direct interaction with 491 interactions from I2D

Tags: cancer

**I2D-MGI-Mouse2Human**	**0.10 %**

Ontological visualization of protein-protein interactions. Drabkin et al. (2005). *BMC Bioinformatics*.

Note: I2D predictions of protein protein interactions using MGI Mus musculus data

Source: Direct interaction with 729 interactions from I2D

**I2D-IntAct-Rat2Human**	**0.02 %**

The IntAct molecular interaction database in 2010. Aranda et al. (2010). *Nucleic Acids Res*.

Note: I2D predictions of protein protein interactions using IntAct Rattus norvegicus data

Source: Direct interaction with 1,055 interactions from I2D

**I2D-MINT-Worm2Human**	**0.02 %**

MINT: a Molecular INTeraction database. Zanzoni et al. (2002). *FEBS Lett*.

Note: I2D predictions of protein protein interactions using MINT Caenorhabditis elegans data

Source: Direct interaction with 1,170 interactions from I2D

**I2D-BIND-Worm2Human**	**0.01 %**

BIND--a data specification for storing and describing biomolecular interactions, molecular complexes and pathways. Bader et al. (2000). *Bioinformatics*.

Note: I2D predictions of protein protein interactions using BIND Caenorhabditis elegans data

Source: Direct interaction with 914 interactions from I2D

**Pathway**	**5.04 %**
**Wu-Stein-2010**	**1.72 %**

A human functional protein interaction network and its application to cancer data analysis. Wu et al. (2010). *Genome Biol*.

Source: 78,183 interactions from supplementary material

Tags: transcription factors; cancer

**PATHWAYCOMMONS-REACTOME**	**1.54 %**

Source: Direct interaction with 24,930 interactions from Pathway Commons

**PATHWAYCOMMONS-NCI_NATURE**	**0.68 %**

Source: Direct interaction with 10,109 interactions from Pathway Commons

**PATHWAYCOMMONS-CELL_MAP**	**0.56 %**

Source: Direct interaction with 587 interactions from Pathway Commons

**PATHWAYCOMMONS-IMID**	**0.51 %**

Source: Direct interaction with 934 interactions from Pathway Commons

**PATHWAYCOMMONS-HUMANCYC**	**0.04 %**

Source: Direct interaction with 685 interactions from Pathway Commons

**Co-localization**	**3.22 %**
**Schadt-Shoemaker-2004**	**1.98 %**

A comprehensive transcript index of the human genome generated using microarrays and computational approaches. Schadt et al. (2004). *Genome Biol*.

Note: Predicted transcript array

Source: Pearson correlation with 60,216 interactions from GEO

**Johnson-Shoemaker-2003**	**1.24 %**

Genome-wide survey of human alternative pre-mRNA splicing with exon junction microarrays. Johnson et al. (2003). *Science*.

Source: Pearson correlation with 426,640 interactions from GEO

Tags: cultured cells; cell line

**Genetic interactions**	**1.68 %**
**BIOGRID-SMALL-SCALE-STUDIES**	**0.48 %**

Source: Direct interaction with 408 interactions from BioGRID

**Lin-Boeke-2012 A**	**0.42 %**

Functional dissection of lysine deacetylases reveals that HDAC1 and p300 regulate AMPK. Lin et al. (2012). *Nature*.

Note: One of 2 datasets produced from this publication.

Source: Direct interaction with 599 interactions from BioGRID

Tags: cell proliferation; transcription factors; cell line; cultured cells; cancer

**Toyoshima-Grandori-2012**	**0.34 %**

Functional genomics identifies therapeutic targets for MYC-driven cancer. Toyoshima et al. (2012). *Proc Natl Acad Sci U S A*.

Source: Direct interaction with 101 interactions from BioGRID

Tags: cancer

**IREF-SMALL-SCALE-STUDIES**	**0.32 %**

Source: Direct interaction with 55 interactions from iRefIndex

**Lin-Boeke-2012 B**	**0.12 %**

Functional dissection of lysine deacetylases reveals that HDAC1 and p300 regulate AMPK. Lin et al. (2012). *Nature*.

Note: One of 2 datasets produced from this publication.

Source: Direct interaction with 266 interactions from BioGRID

Tags: cell proliferation; transcription factors; cell line; cultured cells; cancer

**Shared protein domains**	**0.84 %**
**INTERPRO**	**0.48 %**

Source: Pearson correlation with 562,560 interactions from InterPro

**PFAM**	**0.36 %**

Source: Pearson correlation with 543,464 interactions from Pfam

Attributes

Attribute	Gene

Genes

FOXC2 forkhead box C2 (MFH-1, mesenchyme forkhead 1) [Source:HGNC Symbol;Acc:3801]

Functions:

1 2 3 4 5 6 7 8 9 10 12 15 16 17 18 19 20 21 22 24 27 29 30

Synonyms: ENSG00000176692; ENSP00000326371; 2303; FOXC2; NP_005242; NM_005251; FKHL14; MFH-1; FOXC2_HUMAN; Q99958;

More at Entrez

KDM5B lysine (K)-specific demethylase 5B [Source:HGNC Symbol;Acc:18039]

Functions:

Synonyms: ENSG00000117139; ENSP00000235790; ENSP00000356233; ENSP00000356234; ENSP00000473481; 10765; KDM5B; NP_006609; NM_006618; CT31; JARID1B; PLU-1; RBBP2H1A; KDM5B_HUMAN; Q9UGL1;

More at Entrez

FOXB1 forkhead box B1 [Source:HGNC Symbol;Acc:3799]

Functions:

2 4 5 9 10 19 22 27 30

Synonyms: ENSG00000171956; ENSP00000379369; 27023; FOXB1; NP_036314; NM_012182; FKH5; HFKH-5; FOXB1_HUMAN; Q99853;

More at Entrez

ZNF197 zinc finger protein 197 [Source:HGNC Symbol;Acc:12988]

Functions:

Synonyms: ENSG00000186448; ENSP00000334616; ENSP00000345809; ENSP00000373250; ENSP00000373251; ENSP00000379370; ENSP00000394713; 10168; ZNF197; NP_001020026; NP_008922; NM_001024855; NM_006991; D3S1363E; ZKSCAN9; ZNF166; ZSCAN41; O14709; ZN197_HUMAN;

More at Entrez

PPP2R2A protein phosphatase 2, regulatory subunit B, alpha [Source:HGNC Symbol;Acc:9304]

Functions:

Synonyms: ENSG00000221914; ENSP00000325074; ENSP00000370113; ENSP00000428164; ENSP00000428165; ENSP00000428542; ENSP00000428705; ENSP00000428800; ENSP00000429279; ENSP00000430320; 5520; PPP2R2A; NP_001171062; NP_002708; NM_001177591; NM_002717; B55A; PR52A; PR55A; 2ABA_HUMAN; P63151;

More at Entrez

NEUROG3 neurogenin 3 [Source:HGNC Symbol;Acc:13806]

Functions:

4 13 29

Synonyms: ENSG00000122859; ENSP00000242462; 50674; NEUROG3; NP_066279; NM_020999; Atoh5; bHLHa7; Math4B; ngn3; NGN3_HUMAN; Q9Y4Z2;

More at Entrez

PYY peptide YY [Source:HGNC Symbol;Acc:9748]

Functions:

Synonyms: ENSG00000131096; ENSP00000353198; ENSP00000467310; 5697; PYY; NP_004151; NM_004160; PYY1; P10082; PYY_HUMAN;

More at Entrez

HSF4 heat shock transcription factor 4 [Source:HGNC Symbol;Acc:5227]

Functions:

Synonyms: ENSG00000102878; ENSP00000264009; ENSP00000403219; ENSP00000408815; ENSP00000427832; ENSP00000427963; ENSP00000428077; ENSP00000428161; ENSP00000428978; ENSP00000429486; ENSP00000429580; ENSP00000430211; ENSP00000430299; ENSP00000430631; ENSP00000430724; ENSP00000430840; ENSP00000430947; ENSP00000463706; 3299; HSF4; NP_001035757; NP_001529; NM_001040667; NM_001538; CTM; HSF4_HUMAN; Q9ULV5;

More at Entrez

NLE1 notchless homolog 1 (Drosophila) [Source:HGNC Symbol;Acc:19889]

Functions:

Synonyms: ENSG00000073536; ENSP00000354075; ENSP00000413572; ENSP00000465420; ENSP00000466588; ENSP00000466764; ENSP00000468339; 54475; NLE1; NP_001014445; NP_060566; NM_001014445; NM_018096; FLJ10458; NLE1_HUMAN; Q9NVX2;

More at Entrez

FOXD2 forkhead box D2 [Source:HGNC Symbol;Acc:3803]

Functions:

2 3 4 5 6 7 8 9 10 11 12 13 14 15 16 17 18 19 20 21 22 23 24 25 26 28

Synonyms: ENSG00000186564; ENSP00000335493; 2306; FOXD2; NP_004465; NM_004474; FKHL17; FREAC9; FOXD2_HUMAN; O60548;

More at Entrez

ASGR1 asialoglycoprotein receptor 1 [Source:HGNC Symbol;Acc:742]

Functions:

Synonyms: ENSG00000141505; ENSP00000269299; ENSP00000370307; ENSP00000458530; ENSP00000458803; ENSP00000459169; ENSP00000459896; ENSP00000467511; 432; ASGR1; NP_001184145; NP_001662; NM_001197216; NM_001671; CLEC4H1; ASGR1_HUMAN; P07306;

More at Entrez

EBF2 early B-cell factor 2 [Source:HGNC Symbol;Acc:19090]

Functions:

Synonyms: ENSG00000221818; ENSP00000386178; ENSP00000430241; ENSP00000437909; 64641; EBF2; NP_073150; NM_022659; COE2; FLJ11500; COE2_HUMAN; Q9HAK2;

More at Entrez

MAP2K7 mitogen-activated protein kinase kinase 7 [Source:HGNC Symbol;Acc:6847]

Functions:

Synonyms: ENSG00000076984; ENSP00000381066; ENSP00000381068; ENSP00000381070; ENSP00000443946; 5609; MAP2K7; NP_660186; NM_145185; Jnkk2; MKK7; PRKMK7; MP2K7_HUMAN; O14733;

More at Entrez

TRIM3 tripartite motif containing 3 [Source:HGNC Symbol;Acc:10064]

Functions:

Synonyms: ENSG00000110171; ENSP00000340797; ENSP00000352508; ENSP00000433070; ENSP00000433102; ENSP00000437283; ENSP00000441091; ENSP00000445460; 10612; TRIM3; NP_001234935; NP_001234936; NP_006449; NP_150594; NM_001248006; NM_001248007; NM_006458; NM_033278; BERP; HAC1; RNF22; RNF97; O75382; TRIM3_HUMAN;

More at Entrez

ADRA1A adrenoceptor alpha 1A [Source:HGNC Symbol;Acc:277]

Functions:

Synonyms: ENSG00000120907; ENSP00000276393; ENSP00000346557; ENSP00000351725; ENSP00000369946; ENSP00000369947; ENSP00000369955; ENSP00000369956; ENSP00000369960; ENSP00000369961; ENSP00000430414; ENSP00000430793; ENSP00000431073; 148; ADRA1A; NP_000671; NP_150645; NP_150646; NP_150647; NM_000680; NM_033302; NM_033303; NM_033304; ADRA1C; ADRA1L1; ADA1A_HUMAN; P35348;

More at Entrez

SMR3B submaxillary gland androgen regulated protein 3B [Source:HGNC Symbol;Acc:17326]

Functions:

Synonyms: ENSG00000171201; ENSP00000302400; ENSP00000423138; 10879; SMR3B; NP_006676; NM_006685; P-B; PROL3; P02814; SMR3B_HUMAN;

More at Entrez

FOXD1 forkhead box D1 [Source:HGNC Symbol;Acc:3802]

Functions:

2 3 6 7 8 9 11 12 14 15 16 17 18 20 21 24 28 30

Synonyms: ENSG00000251493; ENSP00000462795; 2297; FOXD1; NP_004463; NM_004472; FKHL8; FREAC4;

More at Entrez

SEC14L4 SEC14-like 4 (S. cerevisiae) [Source:HGNC Symbol;Acc:20627]

Functions:

Synonyms: ENSG00000133488; ENSP00000255858; ENSP00000314163; ENSP00000317617; ENSP00000371412; ENSP00000376525; ENSP00000440848; 284904; SEC14L4; NP_001154840; NP_777637; NM_001161368; NM_174977; dJ130H16.5; TAP3; Q9UDX3; S14L4_HUMAN;

More at Entrez

FOXD3 forkhead box D3 [Source:HGNC Symbol;Acc:3804]

Functions:

2 3 4 5 6 7 8 9 10 11 12 13 14 15 16 17 18 19 20 21 22 23 24 25 26 28 30

Synonyms: ENSG00000187140; ENSP00000360157; 27022; FOXD3; NP_036315; NM_012183; Genesis; HFH2; FOXD3_HUMAN; Q9UJU5;

More at Entrez

NTN3 netrin 3 [Source:HGNC Symbol;Acc:8030]

Functions:

Synonyms: ENSG00000162068; ENSP00000293973; 4917; NTN3; NP_006172; NM_006181; NTN2L; NET3_HUMAN; O00634;

More at Entrez

VGF VGF nerve growth factor inducible [Source:HGNC Symbol;Acc:12684]

Functions:

Synonyms: ENSG00000128564; ENSP00000249330; ENSP00000400884; 7425; VGF; NP_003369; NM_003378; O15240; VGF_HUMAN;

More at Entrez

Functions

Function	FDR	Coverage
1 query genes	n/a	1 / 1
2 DNA binding, bending	4.87E-5	5 / 53
3 ureteric bud development	9.83E-4	4 / 41
4 structure-specific DNA binding	2.38E-3	5 / 142
5 RNA polymerase II distal enhancer sequence-specific DNA binding transcription factor activity	2.43E-3	4 / 66
6 morphogenesis of a branching epithelium	2.43E-3	4 / 67
7 branching morphogenesis of an epithelial tube	2.43E-3	4 / 65
8 morphogenesis of a branching structure	3.12E-3	4 / 74
9 pattern specification process	3.22E-3	5 / 184
10 double-stranded DNA binding	4.04E-3	4 / 84
11 ureteric bud morphogenesis	9.42E-3	3 / 31
12 kidney development	1.24E-2	4 / 117
13 peripheral nervous system development	1.24E-2	3 / 36
14 branching involved in ureteric bud morphogenesis	1.25E-2	3 / 37
15 renal system development	1.27E-2	4 / 125
16 epithelial tube morphogenesis	1.34E-2	4 / 129
17 tube morphogenesis	1.37E-2	4 / 134
18 urogenital system development	1.37E-2	4 / 132
19 transcription factor complex	2.3E-2	4 / 155
20 morphogenesis of an epithelium	2.41E-2	4 / 159
21 tube development	2.91E-2	4 / 169
22 sequence-specific DNA binding RNA polymerase II transcription factor activity	5.24E-2	4 / 199
23 sensory system development	6.2E-2	2 / 12
24 tissue morphogenesis	6.2E-2	4 / 212
25 enteric nervous system development	7.55E-2	2 / 14
26 melanocyte differentiation	7.55E-2	2 / 14
27 somitogenesis	7.55E-2	2 / 14
28 connective tissue development	7.91E-2	3 / 87
29 chromatin DNA binding	8.08E-2	2 / 15
30 sequence-specific DNA binding	9.75E-2	4 / 254

Interactions

Gene 1	Gene 2	Weight	Network group	Networks
NLE1	FOXC2	0.04697827994823456	Co-expression	Bild-Nevins-2006 B<br>Cheok-Evans-2003<br>Rieger-Chu-2004
HSF4	FOXC2	0.04560056887567043	Co-expression	Salaverria-Siebert-2011<br>Wang-Maris-2006<br>Wu-Garvey-2007
EBF2	FOXC2	0.044810318388044834	Co-expression	Roth-Zlotnik-2006<br>Smirnov-Cheung-2009
ASGR1	FOXC2	0.04471665248274803	Co-expression	Bild-Nevins-2006 B<br>Rieger-Chu-2004<br>Wu-Garvey-2007
NEUROG3	FOXC2	0.04470365378074348	Co-expression	Bild-Nevins-2006 B<br>Innocenti-Brown-2011<br>Mallon-McKay-2013
ZNF197	FOXC2	0.04396491311490536	Co-expression	Bild-Nevins-2006 B<br>Burington-Shaughnessy-2008
FOXD2	NEUROG3	0.043765848502516747	Co-expression	Bild-Nevins-2006 B<br>Wu-Garvey-2007
NEUROG3	FOXB1	0.04204022418707609	Co-expression	Gysin-McMahon-2012<br>Innocenti-Brown-2011<br>Mallon-McKay-2013<br>Roth-Zlotnik-2006
SEC14L4	FOXC2	0.04025964066386223	Co-expression	Arijs-Rutgeerts-2009<br>Salaverria-Siebert-2011
PYY	FOXC2	0.0373309226706624	Co-expression	Ramaswamy-Golub-2001<br>Wang-Maris-2006
VGF	NEUROG3	0.03609842713922262	Co-expression	Arijs-Rutgeerts-2009<br>Wu-Garvey-2007
MAP2K7	FOXC2	0.03571854345500469	Co-expression	Burington-Shaughnessy-2008<br>Salaverria-Siebert-2011
VGF	FOXC2	0.03545065503567457	Co-expression	Burington-Shaughnessy-2008<br>Rieger-Chu-2004
TRIM3	FOXC2	0.0341287674382329	Co-expression	Burington-Shaughnessy-2008<br>Salaverria-Siebert-2011
FOXD2	FOXC2	0.032562218606472015	Co-expression	Cheok-Evans-2003<br>Roth-Zlotnik-2006
ADRA1A	FOXC2	0.03058313112705946	Co-expression	Ramaswamy-Golub-2001<br>Salaverria-Siebert-2011
SMR3B	FOXC2	0.029843716882169247	Co-expression	Ramaswamy-Golub-2001<br>Wang-Maris-2006

Gene 1	Gene 2	Weight	Network group	Networks
NTN3	FOXC2	0.029810241423547268	Co-expression	Bild-Nevins-2006 B<br>Ramaswamy-Golub-2001
FOXB1	FOXC2	0.02945294650271535	Co-expression	Innocenti-Brown-2011<br>Mallon-McKay-2013
TRIM3	FOXD2	0.02635238878428936	Co-expression	Kang-Willman-2010
VGF	MAP2K7	0.02525254338979721	Co-expression	Burington-Shaughnessy-2008
SMR3B	FOXD2	0.025134509429335594	Co-expression	Rieger-Chu-2004
TRIM3	ZNF197	0.025032274425029755	Co-expression	Rieger-Chu-2004
FOXD3	FOXD2	0.02435959503054619	Co-expression	Roth-Zlotnik-2006
TRIM3	NEUROG3	0.024228164926171303	Co-expression	Salaverria-Siebert-2011
VGF	FOXB1	0.022132189944386482	Co-expression	Salaverria-Siebert-2011
FOXD1	FOXC2	0.020198279060423374	Co-expression	Innocenti-Brown-2011<br>Roth-Zlotnik-2006
ADRA1A	EBF2	0.01946168951690197	Co-expression	Mallon-McKay-2013
PPP2R2A	KDM5B	0.016437049955129623	Co-expression	Bild-Nevins-2006 B
NTN3	ZNF197	0.016006695572286844	Co-expression	Bild-Nevins-2006 B<br>Burington-Shaughnessy-2008
VGF	ASGR1	0.01595144346356392	Co-expression	Rieger-Chu-2004
FOXD3	FOXC2	0.014324173564091325	Co-expression	Innocenti-Brown-2011<br>Salaverria-Siebert-2011
NTN3	NEUROG3	0.014171199407428503	Co-expression	Roth-Zlotnik-2006<br>Salaverria-Siebert-2011
TRIM3	HSF4	0.012714199721813202	Co-expression	Gysin-McMahon-2012
NTN3	FOXD2	0.011717014014720917	Co-expression	Rieger-Chu-2004
PYY	FOXB1	0.010225965641438961	Co-expression	Roth-Zlotnik-2006
FOXD1	NEUROG3	0.008395100012421608	Co-expression	Innocenti-Brown-2011
FOXD1	EBF2	0.008089721202850342	Co-expression	Roth-Zlotnik-2006
VGF	NTN3	0.0072554415091872215	Co-expression	Gysin-McMahon-2012
FOXD1	HSF4	0.007187672425061464	Co-expression	Mallon-McKay-2013
MAP2K7	NEUROG3	0.005269447807222605	Co-expression	Wang-Maris-2006
NTN3	TRIM3	0.004407675936818123	Co-expression	Wu-Garvey-2007
NTN3	MAP2K7	0.004374683368951082	Co-expression	Bild-Nevins-2006 B
NTN3	SMR3B	0.004280054941773415	Co-expression	Bild-Nevins-2006 B
FOXD3	EBF2	0.004173415247350931	Co-expression	Innocenti-Brown-2011
NTN3	NLE1	0.0035729873925447464	Co-expression	Bild-Nevins-2006 B
FOXD3	NEUROG3	0.0028187583666294813	Co-expression	Innocenti-Brown-2011
ADRA1A	NEUROG3	0.015332119539380074	Co-localization	Johnson-Shoemaker-2003
VGF	NTN3	0.011137884110212326	Co-localization	Johnson-Shoemaker-2003
PPP2R2A	FOXC2	0.44884273409843445	Physical interactions	IREF-INTACT
KDM5B	FOXC2	0.21458150446414948	Physical interactions	BIOGRID-SMALL-SCALE-STUDIES

Gene 1	Gene 2	Weight	Network group	Networks
FOXD2	FOXC2	0.04254680685698986	Shared protein domains	INTERPRO<br>PFAM
FOXD1	FOXC2	0.042508743703365326	Shared protein domains	INTERPRO<br>PFAM
FOXD1	FOXD2	0.042508743703365326	Shared protein domains	INTERPRO<br>PFAM
FOXD3	FOXC2	0.042505837976932526	Shared protein domains	INTERPRO<br>PFAM
FOXD3	FOXD2	0.042505837976932526	Shared protein domains	INTERPRO<br>PFAM
FOXD3	FOXD1	0.04246784932911396	Shared protein domains	INTERPRO<br>PFAM
FOXB1	FOXC2	0.04205899313092232	Shared protein domains	INTERPRO<br>PFAM
FOXD2	FOXB1	0.04205899313092232	Shared protein domains	INTERPRO<br>PFAM
FOXD1	FOXB1	0.04202092997729778	Shared protein domains	INTERPRO<br>PFAM
FOXD3	FOXB1	0.04201802425086498	Shared protein domains	INTERPRO<br>PFAM
NLE1	PPP2R2A	0.005774375982582569	Shared protein domains	PFAM

Search results generated by the GeneMANIA algorithm (genemania.org)
