# Supplementary material for: In silico analysis of single nucleotide polymorphisms (SNPs) in human FOXC2 gene
Source: F1000Res. 2017 Oct 9;6:243. Originally published 2017 Mar 9. [Version 2] doi: 10.12688/f1000research.10937.2 (PMC5814747; doi:10.12688/f1000research.10937.2)
Supplement: Peer review history

# Peer review: Referee response for version 2

**DOI:** 10.5256/f1000research.13959.r26810

**Published:** 2018-02-15

**Authors:** Pascal Brouillard

**Affiliations:** 1 Laboratory of Human Molecular Genetics, de Duve Institute, Université catholique de Louvain, Brussels, Belgium

Most of the remarks have been followed. The only thing left is that this study remains restricted to changes present in dbSNP, in which a number of known mutations are missing. However, I understand that the main goal of this paper is to show a nice bioinformatic analysis strategy rather than to be exhaustive about FOXC2. Thus, it is good as such.

I have read this submission. I believe that I have an appropriate level of expertise to confirm that it is of an acceptable scientific standard.

# Peer review: Referee response for version 2

**DOI:** 10.5256/f1000research.13959.r26811

**Published:** 2017-10-30

**Authors:** Fengkai Zhang

**Affiliations:** 1 Laboratory of Systems Biology, National Institute of Allergy and Infectious Diseases, National Institutes of Health (NIH), Bethesda, MD, USA

Thanks for the authors to address the questions I had in reviewing version 1 of the manuscript.

I have read this submission. I believe that I have an appropriate level of expertise to confirm that it is of an acceptable scientific standard.

# Peer review: Referee response for version 1

**DOI:** 10.5256/f1000research.11789.r22567

**Published:** 2017-05-19

**Authors:** Fengkai Zhang

**Affiliations:** 1 Laboratory of Systems Biology, National Institute of Allergy and Infectious Diseases, National Institutes of Health (NIH), Bethesda, MD, USA

The manuscript presents nice work in analyzing the SNPs in the human FOXC2 gene with a set of publicly available bioinformatics tools and identified 2 nsSNPs and several other UTR region SNPs with potential significance in Lymphedema. The pipeline of tools has been well described and it could benefit research and analysis of other important genes associated with diseases. As the authors pointed out, the findings need further confirmation. I would suggest the authors to address the following aspects: (1) Is there any relationship or coordintation among the identified SNPs? Do they belong to the same haplotype(s)? (2) What is the consideration or motivation to select the tools used for this study? For example, why did the authors choose GeneMANIA? All other tools in this study are used to analyze the SNPs in FOXC2 gene. GeneMANIA gives the result of the network involving FOXC2 with other genes. However, FOXC2 has been well introduced with multiple references at the beginning of this manuscript about its importance in Lymphedema. GeneMANIA does not show obvious help in SNP analysis focused in this study. (3) What is the criteria to identify the positive and negative SNPs for FOXC2, and why? How can false negative be avoided?

 Besides the above major reservations, I would like to ask the authors to clarify/correct the following:

- As pointed by the first referee, what is the total number of SNPs? The abstract and result sections indicated 448 SNPs and the discussion indicated 472 SNPs. How can the two numbers be calculated from 429 nsSNPs and 44 UTR region SNPs (429+44=473)?
- In both abstract and discussion sections, the authors indicate the results require further confirmation. Can the authors describe some possible methods, like experimental, clinical or statistical?
- Page 3, spell out NFATC1 when it appears at the first time
  .
- Page 3, rephrase
  “SNPs are located in vital regions of the gene, which may code for proteins located at the forkhead active domain of the protein or other sites, may severely affect the function of the TF.”
- Page 6, figure 3, the color codes are hard to distinguish in the network visualization. Use alternative method, or give a more detailed explanation.

I have read this submission. I believe that I have an appropriate level of expertise to confirm that it is of an acceptable scientific standard, however I have significant reservations, as outlined above.

# Peer review: Referee response for version 1

**DOI:** 10.5256/f1000research.11789.r21906

**Published:** 2017-04-18

**Authors:** Pascal Brouillard

**Affiliations:** 1 Laboratory of Human Molecular Genetics, de Duve Institute, Université catholique de Louvain, Brussels, Belgium

This paper describes an elegant bioinformatic analysis of variations in a known gene (FOXC2) for primary lymphedema. It uses numerous freely available tools to identify not only variants that can possibly alter the function of the protein, but also affect miRNA (binding) sites. However, one weakness is that it only focuses on changes present in dbSNP, which lacks known amino acid-changing mutations such as e.g. those identified by Van Steenstel et al. or Sholto-Douglas-Vernon et al. ^1^^,^^2^. The results of the PolymiRTS should also be explained a bit more, especially on how to interpret the scores and what to consider as important. Indeed, contrary to the two known amino acids that have been previously characterized, these have not been proven. Focusing on the conservation score is probably not enough because some scores of 2 have a stronger "context+score change" effect than the scores of 7.

 There are several mistakes that need to be corrected:

- The number of SNPs is unclear. The abstract  and results mention a total of 448 and the discussion 472. Yet, neither of these corresponds to 429 nsSNPs + 44 in UTRs (= altogether 474)
  Page 3, right column, second line, SNPs may "code for amino acids", not proteins.
- Page 3, last paragraph, the Biomart link can stop after /martview/. The alphanumerical term after is a changing each time you open a session.
- Page 4, line 10: ... affected BY the mutation ..., not "but"
- Page 4, GeneMANIA is Figure 3, not 2, and PolymiRTS reffers to table 2 to 4, not 3-5.
- The bolded title of Figure 2 cannot be solely rs121909107.
- Page 6, Discussion, 4th line "the mutation leads to TRADUCTION of a histidine ..., not transcription.
- Page 6, right column, the first sentence is strange. It is not the mutation into a leucine at position 125 that means that "each amino acid has its own specific size, charge and hydrophobicity-value". This is a general fact!
- Page 6, right col, line 5: AN amino acid, not as. Line 14: interpro domain. Line 15, DNA in capital letters. Second paragraph, GeneMania links to Figure 3, not 2. Second-last line, refer to Tables 2-4.

I have read this submission. I believe that I have an appropriate level of expertise to confirm that it is of an acceptable scientific standard, however I have significant reservations, as outlined above.

## References

[1] van SteenselMADamstraRJHeitinkMVBladergroenRSVeraartJSteijlenPMvan GeelM: Novel missense mutations in the FOXC2 gene alter transcriptional activity. *Hum Mutat*.2009;30(12) : 10.1002/humu.21127 E1002-9 10.1002/humu.21127 19760751

[2] Sholto-Douglas-VernonCBellRBriceGMansourSSarfaraziMChildAHSmithAMellorRBurnandKMortimerPJefferyS: Lymphoedema-distichiasis and FOXC2: unreported mutations, de novo mutation estimate, families without coding mutations. *Hum Genet*.2005;117(2-3) : 10.1007/s00439-005-1275-2 238-42 10.1007/s00439-005-1275-2 15906099
